# Modulation of B cell activation by extracellular vesicles and potential alteration of this pathway in patients with rheumatoid arthritis

**DOI:** 10.1186/s13075-022-02837-3

**Published:** 2022-07-16

**Authors:** Héctor Rincón-Arévalo, Catalina Burbano, Laura Atehortúa, Mauricio Rojas, Adriana Vanegas-García, Gloria Vásquez, Diana Castaño

**Affiliations:** 1 Grupo de Inmunología Celular e Inmunogenética, Facultad de Medicina, Instituto de Investigaciones Médicas, Universidad de Antioquia UdeA, Calle 70 52-21, Medellín, Colombia; 2 Unidad de Citometría, Facultad de Medicina, Sede de Investigación Universitaria, Universidad de Antioquia UdeA, Calle 70 52-21, Medellín, Colombia; 3 Unidad Reumatología, Hospital San Vicente Fundación, Medellín, Colombia

**Keywords:** B cells, Extracellular vesicles, Rheumatoid arthritis, B cell activation, Immune complexes, Autoimmunity, Antibody production, Macrophages

## Abstract

**Background:**

Extracellular vesicles are involved in the intercellular communication of the immune system. In rheumatoid arthritis (RA), these structures are considered a source of autoantigens that drive proinflammatory responses of innate immune cells. A high concentration of circulating medium/large size extracellular vesicles (m/lEVs) and m/lEVs forming immune complexes (m/lEV-ICs) have been associated with disease activity and systemic inflammation in patients with RA. B cells are central components of RA immunopathology because of their involvement in the production of autoantibodies, antigen presentation, and cytokine production. However, the effect of m/lEVs on B cell function in the context of RA and other autoimmune diseases remains unknown.

**Methods:**

We evaluated the effect of m/lEVs obtained from healthy donors (HD) and patients with RA on B cell responses in vitro. In addition, we evaluated the effect of pre-exposition of monocyte-derived macrophages (MDM) to m/lEVs on activation of autologous B cells from HD and patients.

**Results:**

The presence of m/lEVs reduced the frequency of CD69^+^ and CD86^+^ B cells from HD activated by an agonist of antigen receptor. This regulation of the B cell activation markers by m/lEVs was partially dependent on phosphatidylserine binging. These m/lEVs also reduced the proliferation, calcium mobilization, and global phosphorylation of tyrosine. Similar responses were observed in B cells from patients with RA. However, the presence of m/lEVs promoted high antibody levels in B cells cultured with T cell-dependent stimuli by 7 days. In addition, despite the direct inhibitory effect of m/lEVs on early B cell responses, when B cells were cocultured with autologous MDM previously exposed to m/lEVs or m/lEV-ICs, an increased frequency of CD69^+^ B cells from patients with RA was observed, albeit not with cells from HD.

**Conclusions:**

These data together suggest that m/lEVs have a direct modulatory effect in early responses of B cells through B cell receptor that can potentially fail in patients with RA because of the impact of these vesicles over cells of the innate immune system. This phenomenon can potentially contribute to the loss of tolerance and disease activity in patients with RA.

**Supplementary Information:**

The online version contains supplementary material available at 10.1186/s13075-022-02837-3.

## Background

Rheumatoid arthritis (RA) is a systemic autoimmune disease characterized by chronic inflammation of the synovial tissues that can cause irreversible damage to the joints if not treated properly [1]. B cells are central components of RA immunopathology, mainly because of the production of autoantibodies. Anti-cyclic citrullinated protein antibodies and rheumatoid factor are the primary autoantibodies associated with RA [2]. In addition, other abnormal B cell responses have been associated with RA, such as antigen presentation and cytokine production [3–5].

Extracellular vesicles (EVs) are structures naturally released from the cell that are delimited by a lipid bilayer and cannot replicate; however, the generation of EVs increases under physiological conditions including cellular activation and different cell death processes, such as apoptosis, necroptosis, NETosis, and pyroptosis [6, 7]. Medium/large size extracellular vesicles (m/lEVs) contain a variety of components from their parental cells and can participate in different processes, such as intercellular communication, cellular homeostasis, and innate immunity [8–10]. A higher concentration of m/lEVs has been described in the plasma of patients with RA when compared with that of healthy donors (HD) [11, 12]. The source of circulating m/lEVs is diverse in RA patients, as the majority is derived from platelets and leukocytes [13]. The increased count of these structures in the blood has been associated with disease activity measured by the DAS28 score [11, 12] and systemic inflammation according to the high serum levels of IL-6, IL1β, and TNF-α [13]. Similarly, a higher number of m/lEVs has been reported in the synovial fluid of patients with RA when compared to patients with osteoarthritis or reactive arthritis [14].

m/lEVs have been reported as sources of autoantigens and immune complexes (ICs) in several autoimmune diseases, such as systemic lupus erythematosus [15–17]. Of special interest for RA, m/lEVs from these patients showed higher content of citrullinated peptides and the formation of IgG and IgM ICs when compared with those from HD [13, 15]. It has been reported that m/lEVs isolated from the synovial fluid of patients with RA induce the production of BAFF and other cytokines (including IL-6 and IL-8) by fibroblast-like synoviocytes [18]. In addition, monocytes from HD exposed to platelet-derived m/lEVs increased the production of IL-1β, TNF-α, IL-6, and IL-8 when compared with the absence of these vesicles; this effect was even greater with the exposure of m/lEVs forming ICs (m/lEV-ICs) [19]. These data suggest that m/lEVs and, especially, m/lEV-ICs can promote the proinflammatory responses in RA.

However, the effect of m/lEVs on B cells in the context of RA and other autoimmune diseases remains unknown. Previously, it was described that B cells can interact and internalize m/lEVs [20]. Different components of m/lEVs can explain this interaction and can potentially tune B cell responses, such as nucleic acids (DNA, RNA, and miRNA [21, 22]), phosphatidylserine (PS) [15], adhesion molecules (ICAM1, CD11b, and CD62L [23, 24]), citrullinated peptides [13, 15], ICs [6, 15], and alarmins [13, 15], among others [6, 13, 15, 25]. These components may bind different molecules in B cells, including B cell receptor (BCR), Toll-like receptors (TLRs), and low-affinity Fc gamma receptor IIb (FcγRIIb, CD32b). We proposed that m/lEVs from patients with RA can contribute to B cell dysfunction in this disease by promoting their activation.

In this study, we evaluated the effects of m/lEVs and m/lEV-ICs from patients with RA on B cell responses, including calcium mobilization, global tyrosine phosphorylation, the expression of activation markers, proliferation, and cytokine production. Surprisingly, we observed that the direct presence of m/lEVs and m/lEV-ICs suppresses early B cell responses induced by BCR triggering. In contrast, high levels of IgG and IgM were observed in longer cultures after a T cell-dependent activation of B cells in the presence of m/lEVs and m/lEV-ICs. In addition, increased expression of activation markers were observed when B cells from patients with RA were cocultured with autologous monocyte-derived macrophages (MDM) previously exposed to m/lEVs, and, especially, when these structures formed ICs. Similar responses were observed only with supernatants of MDM exposed to m/lEV-ICs. These results suggest that m/lEVs have a direct regulatory effect over B cells through the BCR activation that seems to be not efficient for T cell-dependent stimuli, and in patients with RA because of the impact of these vesicles over cells of the innate immune system.

## Materials and methods

### Patients and controls

Patients with RA were diagnosed according to the American College of Rheumatology and European League Against Rheumatism criteria [26]. HD of similar gender and ages than patients were also included in this study as controls. The samples were collected at the Rheumatology Unit, Hospital Universitario San Vicente Fundación (HUSVF, Medellin, Colombia) during 2018 − 2019. The main demographic and clinical data of patients and HD were obtained from the medical records and personal interviews (Table 1). All patients and controls signed an informed consent previously approved by the ethics committee from the Instituto de Investigaciones Médicas (Facultad de Medicina, Universidad de Antioquia, Medellin, Colombia) and HUSVF. The regulations regarding work with potentially contaminated biological samples from our country and institution were followed, both for their handling and their final discard.

**Table 1:** Demographic and clinical characteristics of patients with RA

	**HD**	**RA**
***n***	19	13
Age^a^	49 (35 − 53)	61 (53 − 63)
Female	17/19	12/13
DAS28^a^	n.a	2.20 (2.00 − 2.94)
CCP3 IgG U/mL^a^	n.a	395 (37 − 1042)
RF IgM U/mL^a^	n.a	21 (4 − 51)
**Therapies**		
Methotrexate	n.a	3/13
Prednisolone	n.a	2/13
Leflunomide	n.a	3/13
Chloroquine	n.a	4/13
Deflazacort	n.a	2/13
Sulfasalazine	n.a	2/13

^a^Median (interquartile range).* n.a.* Not applicable

### Extracellular vesicles

m/lEVs were isolated from the anticoagulated plasma of HD and RA patients with 3.2% buffered sodium citrate solution (Becton Dickinson, CA) [27]. Briefly, leukocytes were removed immediately after collection by centrifugation at 1800 × *g* for 10 min at room temperature (RT, 21 − 23 °C). The 90% upper plasma was then collected and centrifuged at 3000 × *g* for 20 min at RT to remove platelets. Platelet-poor plasma was mixed with phosphate-buffered saline (PBS; Gibco, CA) at the relation of EVs: PBS of 7:10. Then, aliquots of a maximum of 1 mL in 1.5 mL vials were centrifugated at 16,900 × *g* for 60 min at RT. The 90% upper supernatant of every aliquot was washed out and the enriched m/lEVs (in the 10% bottom) were directly frozen in PBS at − 70 °C.

m/lEVs were characterized with the FSC-A and SSC-A parameters of the LSRFortessa flow cytometer and FACS DIVA software (Becton Dickinson) using reference polystyrene spheres of known sizes (0.1, 1, 3, and 6 µm, Polysciences, PA), different cellular populations (monocytes, lymphocytes, granulocytes, and platelets), and PBS to determinate the electronic noise. In addition, some samples of m/lEVs were analyzed through scanning transmission electron microscopy, as previously described [13]. To ensure the vesicular nature, m/lEVs were stained with anti-human CD41a monoclonal antibody (Table S1) and exposed to 0.05% Triton X-100 (Sigma-Aldrich, MO).

m/lEVs used in this study were obtained from pools prepared after mixing three different donors of the respective HD and RA patient study groups. The IC threshold was previously reported following the distribution of circulating m/lEV-ICs in HD and patients with RA [13]. To optimize the formation of ICs, thawed m/lEVs from patients with RA (PRA-m/lEV-ICs) were incubated with 15 µg/mL purified IgG at 37 °C for 60 min. IgG was isolated from pooled serum samples of 16 patients with seropositive RA using the Nab Protein G Spin Kit (Thermo Fisher Scientific, MA) and following the manufacturer’s instructions. IgG preparation from pooled serum contained 286.3 units of anti-CCP (cyclic citrullinated peptide antibody; CCP3 IgG ELISA, Inova diagnostics, CA) [28]. After incubation, m/lEVs were washed with 1 mL of PBS at 16,900 × *g* for 60 min at RT. The formation of ICs was evaluated by staining with F(ab’)2 fraction anti-IgG Alexa Fluor 488 (Table S1) at 4 °C for 30 min in the dark. Then, m/lEVs were washed and analyzed by flow cytometry and the FlowJo vX software (Tree Star, CA). For some experiments, m/lEVs of the patients and controls were incubated with Annexin V PE (Beckton Dickinson) in the presence of Annexin-binding buffer for 20 min at RT, as previously described [27], and then evaluated by flow cytometry.

To estimate the number of m/lEVs per sample, frozen aliquots were thawed at room temperature. Then, 10 µL of m/l EVs in 190 µL of filtered PBS were acquired by flow cytometry (under constant flow and low pressure) until all 200 µL were acquired. The number of m/lEVs in every sample corresponded to the total counts detected inside the m/lEVs gate with a lower limit defined by the electronic noise (according to the acquisition of filtered PBS), and after the correction regarding the dilution used.

### Isolation of B cells

B cells were isolated from EDTA-anti-coagulated blood using rosettes (Stem Cell Technologies, Canada) and following the manufacturer’s instructions. Briefly, after 20 min of incubation with rosette reagent at RT, the samples were diluted with PBS (1:1) and separated on Histopaque (Sigma-Aldrich) at a ratio of 7:3 (Blood-PBS-Rosette: Histopaque), 1200 × *g*, 20 min, and RT. The B cell layer was removed and washed twice with PBS at 300 × *g* for 10 min. The cell number and viability (> 98%) were estimated by Neubauer chamber and trypan blue exclusion (Sigma-Aldrich), respectively. The purity was checked by flow cytometry as CD19^+^ cells (> 85%, Table S1). Freshly isolated B cells were used immediately for staining or culture.

### Flow cytometry

B cells were incubated with blocking buffer [PBS containing 10% inactivated fetal bovine serum (iFBS, Gibco), 0.1% bovine serum albumin (BSA, Sigma-Aldrich), and 0.01% sodium azide (Sigma-Aldrich)] for 15 min at 4 °C. The cells were stained with fluorescently labeled and primary anti-human monoclonal antibodies (Table S1) in PBS for 30 min at 4 °C in the dark. The cells were then washed and immediately analyzed using the LSRFortessa flow cytometer and the FACS DIVA software (Becton Dickinson).

### B cell culture

B cells (1 × 10^5^ cells) were cultured in 96-well U-bottom plates at 37 °C and 5% CO_2_ with 200 µL of complete medium [RPMI 1640 GlutaMAX (Gibco), 10% iFBS, 2 mM L-glutamine (Gibco), 1% streptomycin/penicillin, and 10 ng/mL IL-2 (Biolegend, CA)]. The B cell activation was accomplished with 2.5 µg/mL anti-human IgM/IgG F(ab)_2_ fraction (Jackson ImmunoResearch, PA) for 24 h. In addition, the B cells were stimulated with 2.5 µg/mL anti-human IgM/IgG F(ab)_2_ fraction plus 2.5 µg/mL CpG (Invivogen, CA) for 24 and 72 h. The cultures were performed in the presence or absence of every type of m/lEVs pool at a B cell: m/lEVs ratio of 1:3 vesicle: cell. This ratio was previously described for patients [13, 27], according to the counts of total peripheral blood mononuclear cells (PBMCs) and total m/lEVs. To achieve the ratio of 3:1 vesicle:cell, the volume of m/lEVs was adjusted diluting with RPMI. After culture, the cells were washed with cold PBS and exposed to Live/Dead Fixable Blue Dead Cell Stain (Live/Dead, Thermo-Fischer Scientific, MA) as per the manufacturer’s instructions and then labeled with anti-human monoclonal antibodies for the linage marker CD19 and the activation markers CD69, CD80, and CD86, as described earlier (Table S1).

To determine the dependence of FcγRIIb (CD32b) in B cell responses to m/lEVs and m/lEV-ICs, these cells were incubated with 20 µg/mL anti-human FcγRIIb (clone AT10) for 15 min before the addition of vesicles. The blocking role of AT-10 antibody has been previously reported [29], and we also confirmed its effect by reduction of tyrosine phosphorylation in B cells from HD (Data not shown). To assess the sialic acid-mediated binding, m/lEVs were treated with 100 µg/mL *Sambucus nigra* lectin agglutinin I (SNA-I, EYlabs, CA) or 100 µg/mL *Maackia amurensis* lectin agglutinin (MAA, EYlabs) for 30 min at RT before culturing. To determine the potential role of PS-mediated binding, m/lEVs were incubated with Annexin V as described earlier. The potential contribution of these pathways to B cell modulation by m/lEVs and m/lEV-ICs was evaluated as frequencies of CD69^+^, CD80^+^, and CD86^+^ B cells.

For proliferation, 1 million B cells were stained with 1 mL of 5 µM CellTrace (Violet Cell Proliferation Kit, Thermo Fisher Scientific) in PBS for 15 min at 37 °C. Then, the cells were incubated with 1% BSA in PBS for 30 min at 37 °C to eliminate the excess of CellTrace. B cells were cultured in complete media for 72 h, as previously explained, and the frequency of the proliferating cells and division index were evaluated. The supernatants of these cultures were collected and stored at − 20 °C to measure the cytokine levels.

### Calcium mobilization

The B cells (1 × 10^5^ per condition) were incubated with 2 µg/mL Indo 1 AM calcium dye (Thermo Fisher Scientific) and 0.045% Pluronic F-127 (Invitrogen, CA) in complete medium for 15 min at 37 °C. The cells were washed with RPMI 1640 and stained with anti-human CD19, CD21, CD24, CD27, CD38, and IgM monoclonal antibodies (Table S1) for 15 min at RT. These markers allowed us to divide the B cells according to the memory and naive subsets. Then, the B cells were washed again and suspended in a 450-µL complete medium and maintained at 37 °C for 10 min [30]. The cells were analyzed by flow cytometry for 30 s before and 210 s after the treatment with 10 µg/mL polyclonal anti-human IgM/IgG F(ab)_2_ fraction in the presence or absence of each type of m/lEVs (B cell: m/lEVs ratio of 1:3). As a positive control, the cells were stimulated with 1 µg/mL ionomycin (Sigma-Aldrich) at the end of the acquisition. The relative concentration of intracellular free calcium was calculated based on the median fluorescence intensity (MFI) ratio of Indo 1 bound/unbound detected at 405/485 nm, respectively.

### Global tyrosine phosphorylation

The B cells (1 × 10^5^ cells/mL per condition) were resuspended in a complete medium without serum and incubated for 10 min at 37 °C. Then, the B cells were stimulated with 10 µg/mL polyclonal anti-human IgM F(ab)_2_ fraction for 45 s in the presence or absence of each type of m/lEVs pool (B cell: m/lEVs ratio of 1:3). The cells were placed on an ice bath for 15 min and labeled with anti-human CD19 and CD38 monoclonal antibodies (Table S1). These cells were then fixed and permeabilized with the Foxp3/Transcription Factor Staining Buffer Set Kit (eBiosciences), as previously reported for phosphoproteins [31]. The B cells were labeled with anti-phosphotyrosine antibody (Table S1) in permeabilization buffer at 4 °C and incubated in the dark for 30 min. Pervanadate was used as the positive control of phosphorylation [32]. Fluorescence minus one was used as the staining control. The results obtained were presented as MFI.

### Plasma cell differentiation and antibody production

Enriched B cells from HD and patients with RA (2 × 10^6^ cells/mL) were cultured in 96 U-bottom plates for 7 days in complete medium with 10 ng/mL IL-2, 50 ng/mL IL-21 (Biolegend), and 1 µg/mL CD40L (R&D Systems, MN). Cells were spun down and supernatants were collected and stored at − 20 °C. Cells were stained with Live/Dead following the manufacturer’s instructions, then blocked, labeled with anti-human CD19, CD27, and CD38 monoclonal antibodies, and analyzed by flow cytometry. The results of plasmablast cell differentiation are presented as the frequency of CD19^+^CD27^+^CD38^hi^ cells in total live cells. IgM and IgG levels were measured in supernatants of these cultures by using an enzyme-linked immunosorbent assay (ELISA) Ready-Set-Go! Kit (eBiosciences), following the manufacturer’s instructions.

### Coculture of B cells and MDM

PBMCs were isolated by using the Histopaque-1077 density gradient and defibrinated venous blood samples from HD and patients with RA by centrifugation at 900 × *g* for 30 min at RT. PBMCs containing 1.2 × 10^5^ CD14^+^ cells were cultured in 96-well, flat-bottom and culture-treated plates (Corning, MA), with 250-μL RPMI 1640 GlutaMAX containing 0.5% inactivated autologous serum depleted of m/lEVs (after centrifugation at 16,900 × *g* for 1 h, SDE) for 4 h at 37 °C in 5% CO_2_. To remove the non-adherent cells, the wells were washed multiple times with prewarmed 0.5% SDM in PBS.

The adherent cells were differentiated to MDM by culturing them with 250-μL RPMI 1640 GlutaMAX containing 10% SDM for 6 days at 37 °C under 5% CO_2_ condition [33]. MDMs were treated with PRA-m/lEVs or PRA-m/lEV-ICs (Cell: m/lEVs ratio of 1:3) for 6 h. Then, the cells were washed thoroughly with prewarmed 0.5% SDE in PBS to remove the non-internalized vesicles, and a new medium was added. Autologous B cells were isolated as detailed earlier and suspended in the same medium of culture described for adherent cells, followed by immediately adding it to pre-washed MDM at a ratio of B cells: MDM ratio of 1-2:1. The cocultures were incubated for 96 h at 37 °C under 5% CO_2_.

For some experiments, B cells were cultured alone in the presence of the supernatant of MDM treated or not with PRA-m/lEV-ICs for 6 h. The excess of the vesicles in the supernatant was removed by centrifugation (at 16,900 × *g* for 1 h); only the 50% upper part of the media was used. Monocultures of B cells were incubated for 96 h at 37 °C under 5% CO2. At the end of the culture period, the non-adherent cells were labeled with anti-human CD19, CD69, CD80, and CD86 monoclonal antibodies (Table S1). The samples were immediately analyzed by flow cytometry, and the data were presented as frequencies.

### Monocyte and MDM culture with m/lEVs and m/lEV-ICs

MDM from HD and patients with RA were obtained as explained before. Monocytes were isolated from 50 mL of venous blood from HD using Monocyte Enrichment Cocktail (Stem Cell Technologies) following the manufacturer’s instructions. Total monocytes (purity > 90%) were stained with CD14-RD1 (clone 322A-1; Beckman Coulter, CA) and CD16-FITC (clone 3G8; BD) antibodies for 15 min at 4 °C. CD16 − (purity > 95%) and CD16 + (purity > 90%) monocytes were sorted by using MoFlo XDP (Beckman Coulter, CA).

MDM were cultured for 6 h in the absence or presence of PRA-m/lEVs or PRA-m/lEV-ICs at a ratio monocyte:m/lEVs of 1:3. Enriched monocytes were cultured for 24 h as previously described [34], in the absence or presence of PRA-m/lEVs or PRA-m/lEV-ICs at ratio monocyte:m/lEVs of 1:3. Supernatants were stored at − 20 °C.

### Cytokine measurements

The human Inflammatory Cytometric Bead Array Kit (Becton Dickinson) was used to evaluate the IL-8, IL-6, IL-10, TNF-α, and IL-1β levels by flow cytometry as per the manufacturer’s instructions, in the supernatants of B cells cultured with 2.5 µg/mL anti-human IgM/IgG F(ab)_2_ fraction and 2.5 µg/mL CpG for 24 and 72 h, as well as in supernatants of monocyte cultures and monocultures of MDM in presence or absence of m/lEVs and m/lEV-ICs.

### Data analysis

Flow cytometry analysis including gate definition, cell frequencies, MFI, percentage of divided B cells, and index of the division was calculated using the FlowJo vX software. Comparisons between the cultured B cells with and without m/lEVs were performed using the Kruskal − Wallis test and Dunn’s post-test. B cells cocultured with MDM previously exposed or not to m/lEVs were analyzed with a two-way analysis of variance (ANOVA) test with Šidák post-test. Statistical analysis of B cells cultured in the presence or absence of different inhibitors was performed by using the Friedman test with the Dunn post-test. Analysis of B cells cultured with supernatants of MDM was performed with the Wilcoxon test. All statistical analyses were performed in Prism with the 6.0 software (GraphPad, CA), and *p* < 0.05 was considered to be statistically significant (* *p* < 0.05, ** *p* < 0.01, *** *p* < 0.001, **** *p* < 0.0001). Comparisons were conducted among all the study groups; however, the data of only those that were statistically significant are shown in this paper.

## Results

### Characterization of m/lEVs and m/lEV-ICs

**Fig. 1:**
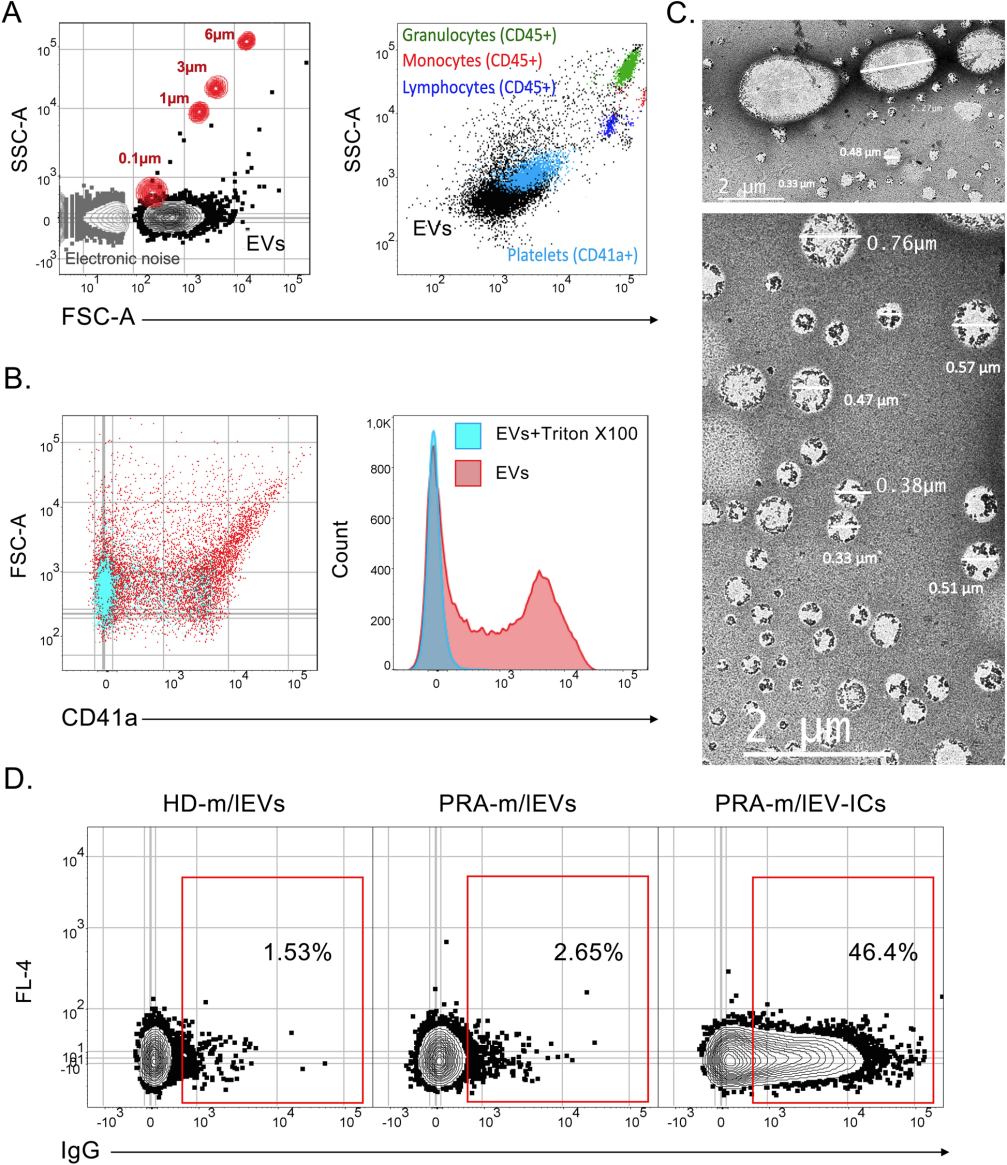
Characterization of the extracellular vesicles (EVs) for B cell treatment. **A** Representative plots of EV size according to the FSC-A and SSC-A parameters defined by using beads with reference diameters (0.1, 1, 3, and 6 µm) (left panel) or granulocyte, monocyte lymphocyte, and platelet populations (right panel). **B** EVs are sensitive to detergent treatment. Representative CD41a versus FSC-A dot plots and CD41a histograms of EVs untreated and treated with 0.05% Triton X-100. CD41a positivity was rapidly altered after Triton X-100 addition. **C** The vesicular nature and size of EVs. Representative microphotographs of scanning transmission electron microscopy of EVs. These images demonstrate the vesicular shape and size expected for m/lEVs. **D** Representative IgG expression of m/lEVs from HD (HD-m/lEVs), patients with RA (PRA-m/lEVs), and those from patients with RA that form immune complexes (PRA-m/lEV-ICs). Representative results of at least three independent experiments are shown for all the experiments

Most of the EVs had a size between 0.1 and 1 µm, using reference polystyrene spheres of known sizes (0.1, 1, 3, and 6 µm), different cellular populations (monocytes, lymphocytes, granulocytes, and platelets), and PBS to determinate the electronic noise (Fig. 1A). In addition, EVs showed a vesicular nature because of the absence of CD41a expression in the presence of Triton X-100 (Fig. 1B). The size and vesicular structure were verified through scanning transmission electron microscopy (Fig. 1C), showing that most of the extracellular vesicles analyzed (> 80%) displayed vesicular shape and expected sizes of 0.1–1 μm; according to Minimal Information for Studies of Extracellular Vesicles 2018 (MISEV2018) guidelines [35], these EVs were hence called m/lEVs in this study.

Three different pools of m/lEVs were included in the experiments according to the study groups (HD and patients) and the formation of ICs: EVs with low frequency of IC formation (m/lEV-ICs, IgG +  < 6%) from 3 HD (HD-m/lEVs, 1.73% ± 2.17) and from 3 patients with RA (PRA-m/lEVs, 1.93% ± 1.13); another pool of m/lEVs from three patients with RA with ICs > 28% was also included in this study (PRA-m/lEV-ICs, 50.40% ± 23.85) (Fig. 1D). The IC threshold was previously reported following the distribution of circulating m/lEV-ICs in HD and patients with RA [13].

### m/lEVs reduce the frequency of CD69 + and CD86 + B cells of healthy controls stimulated in vitro

**Fig. 2:**
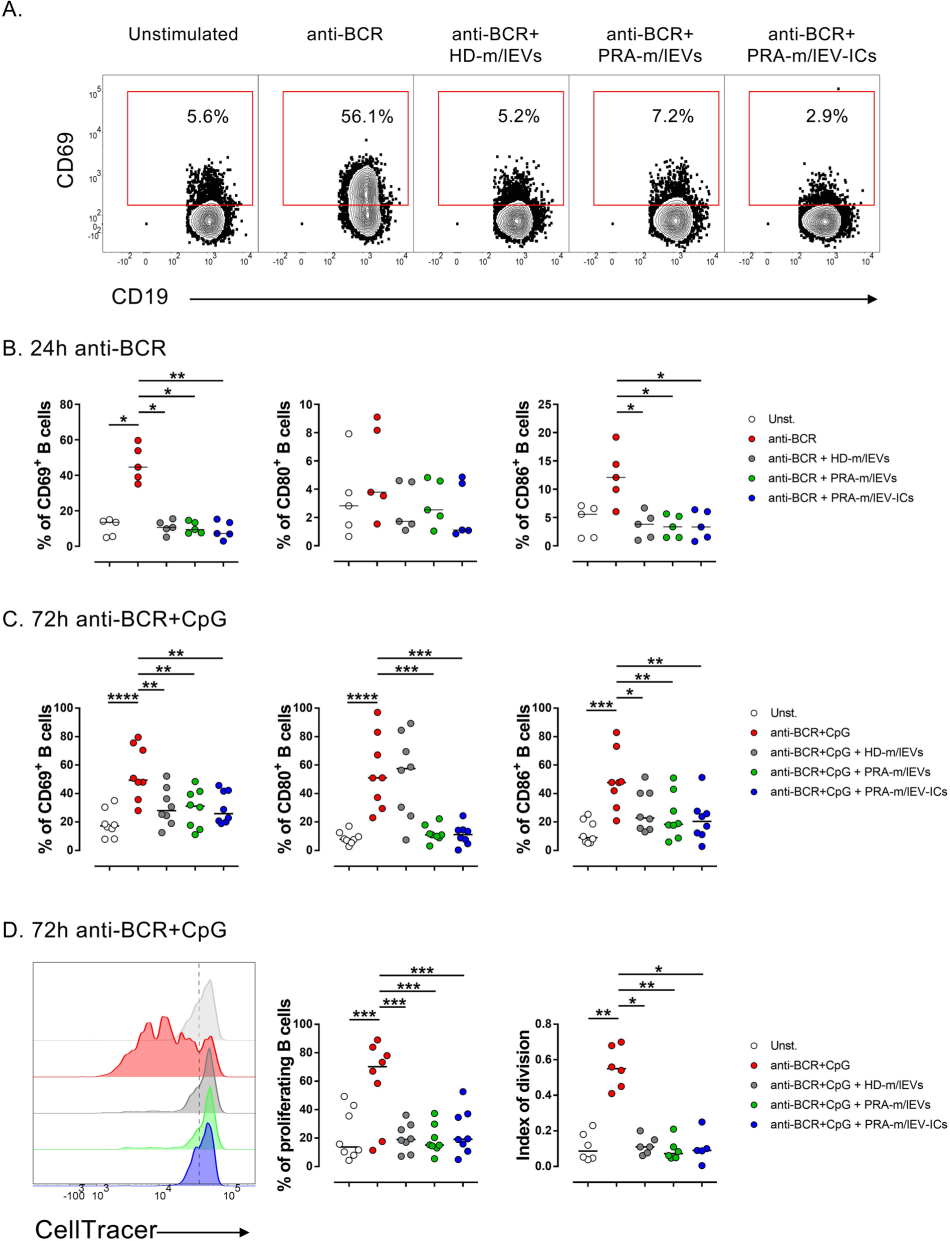
Low frequency of CD69^+^ and CD86^+^ cells and proliferation of in vitro*-*activated B cells exposed to m/lEVs. **A** Representative contour plots of CD69 expression in B cells from HD cultured for 24 h without stimulation (unstimulated, Unst) or with anti-BCR in the absence or presence of m/lEVs from HD (HD-m/lEVs), patients with RA (PRA-m/lEVs), and patients with RA-forming immune complexes (PRA-m/lEV-ICs). **B** Frequency of CD69^+^ (left panel), CD80^+^ (center panel), and CD86^+^ (right panel) B cells, cultured for 24 h without stimulation or with anti-BCR in the absence or presence of HD-m/lEVs, PRA-m/lEVs, or PRA-m/lEV-ICs. The data of five HD donors and medians are shown. **C** The frequency of CD69^+^ (left panel), CD80^+^ (center panel), and CD86.^+^ (right panel) B cells, cultured for 72 h without stimulation or with anti-BCR and CpG in the absence or presence of HD-m/lEVs, PRA-m/lEVs, or PRA-m/lEV-ICs. The data of eight HD donors and medians are shown. **D** Representative histogram of CellTracer expression on B cells cultured for 72 h (left panel), consolidated data of proliferating B cells (center panel), and the index of division (right panel) of B cells cultured for 72 h without stimulation or with anti-BCR + CpG in the absence or presence of HD-m/lEVs, PRA-m/lEVs, or PRA-m/lEV-ICs. Data of 6 − 8 HD donors and median are shown. **B–D** Kruskal − Wallis test with Dunn’s post-test. * *p* < 0.05, ** *p* < 0.01, *** *p* < 0.001, **** *p* < 0.0001

Previously, it was reported that circulating m/lEVs from patients with RA exhibit citrullinated peptides and other components from their parental cells, including alarmins, nucleic acids, and antibodies [13, 19]. All these molecules can potentially modulate B cell responses. To evaluate whether m/lEVs alone can provide any stimuli on their own, the B cells from HD were exposed to m/lEVs from HD (HD-m/lEVs), patients with RA (PRA-m/lEVs), and m/lEVs from patients with RA-forming ICs (PRA-m/lEV-ICs). The presence of m/lEVs neither induced the expression of CD69, CD80, or CD86 in the B cells nor affected the viability of these cells (Fig. S1A). These results together suggested that m/lEVs cannot activate B cells by themselves; however, m/lEVs may modulate B cell responses in the presence of a BCR agonist. Therefore, B cells from HD were stimulated with anti-IgG/IgM (anti-BCR) in the presence or absence of m/lEVs pools (Fig. 2A). No significant changes in CD80^+^ B cells were observed with the anti-BCR stimulus (Fig. 2B). Surprisingly, a decreased proportion of CD69^+^ and CD86^+^ B cells was observed in the presence of HD-m/lEVs, PRA-m/lEVs, or PRA-m/lEV-ICs when compared with the BCR stimulation in the absence of m/lEVs (Fig. 2B). Also, reduced expression of CD40 was observed of anti-BCR stimulated B cells in presence of PRA-m/lEVs compared with the absence of these vesicles (data not shown).

To determine whether the effect of m/lEVs in the expression of activation markers can still be observed after the stimulus of B cells through BCR together with a coreceptor, the B cells were cultured with anti-BCR combined with CpG for 24 h (Fig. S1B) and 72 h (Fig. 2C). No significant changes in the frequency of CD69^+^ and CD86^+^ B cells were observed in the presence of m/lEVs at 24 h when each m/lEV pool was independently evaluated (Fig. S1B); however, a significant decline in CD69^+^ B cells was detected when all m/lEVs were analyzed together (Fig. S1C). After 72 h of culture, a significant reduction in the frequency of CD69^+^, CD80^+^, and CD86^+^ B cells with different m/lEVs when compared with that in the absence of these m/lEVs was noted (Fig. 2C). These results were also observed when all m/lEV pools were analyzed together (Fig. S1C).

To eliminate the competition effect of m/lEVs over B cell agonists, PRA-m/lEVs were added to the B cell culture 1 h before, at the same time, or 1 h after stimulation with anti-BCR plus CpG. The reduction in the frequency of CD69^+^ B cells was still observed with PRA-m/lEVs at all conditions tested after 72 h of culture (Fig. S1D). These data suggest that the modulatory effect of m/lEVs in B cell activation is not the consequence of competition for B cell stimuli.

These results together revealed that m/lEVs of different sources could inhibit the expression of molecules associated with the activation of B cells from HD.

### m/lEVs reduced proliferation, calcium mobilization, and global phosphorylation of tyrosine in B cells of HD stimulated in vitro and ex vivo

To establish whether m/lEVs could modulate other aspects of B cell activation, the levels of secreted cytokines and proliferation of B cells from HD were evaluated after anti-BCR and CpG treatments. Although the difference was not significant, a tendency to lower the concentration of IL-6, IL-10, and TNF-α in the supernatants of B cells with the presence of m/lEVs pools was noted when compared with those without vesicles at both 24 and 72 h of culture (Fig. S2A-B). Decreased frequency of proliferating cells and a lower index of division was observed in B cells exposed to HD-m/lEVs, PRA-m/lEVs, or PRA-m/lEV-ICs when compared with cells cultured in the absence of these EVs at 72 h (Fig. 2D).

**Fig. 3:**
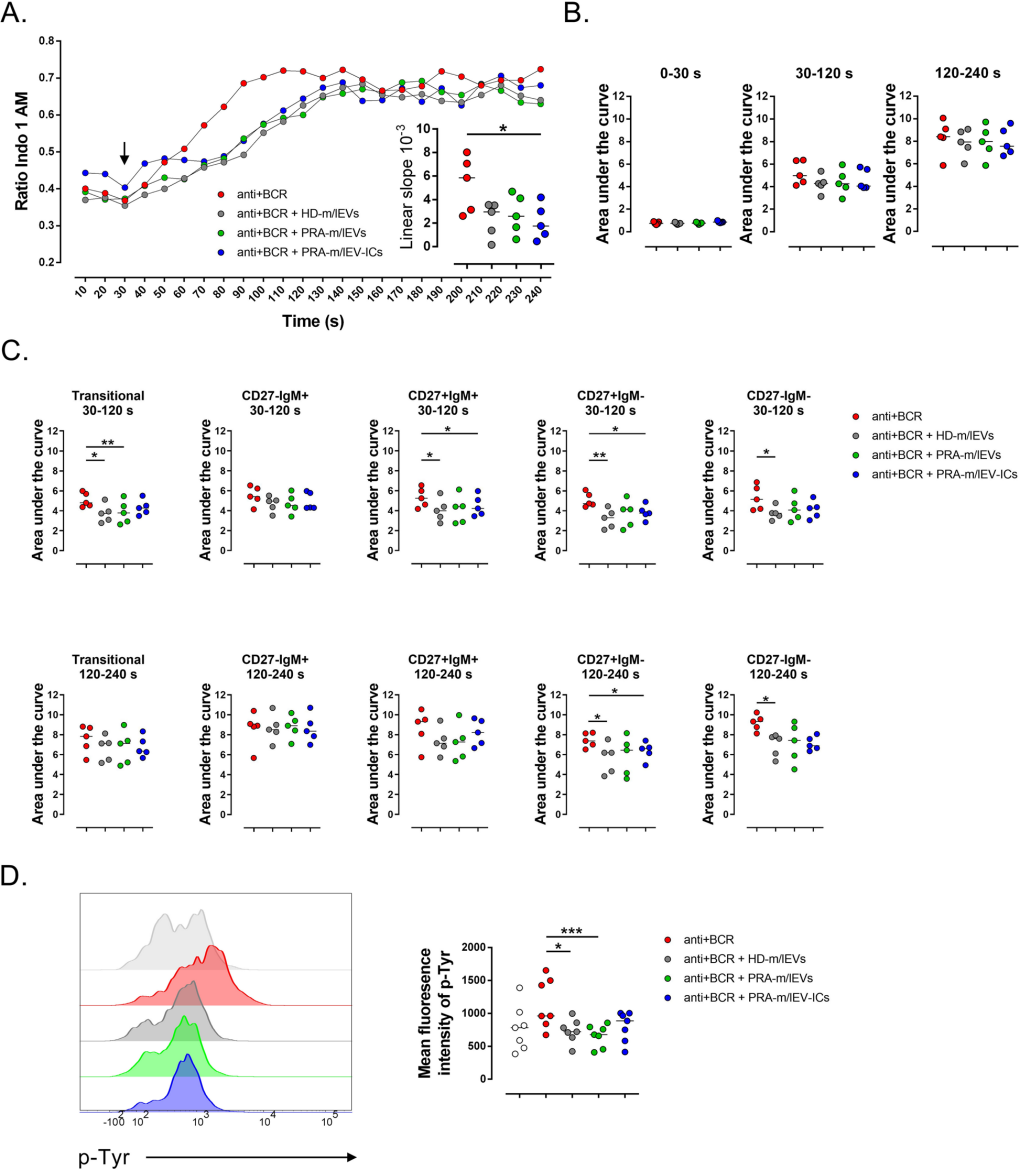
Less calcium mobilization of activated B cells exposed to m/lEVs. **A** The kinetic of calcium mobilization in total B cells based on the ratio of Indo 1 AM bound/unbound. The B cells were kept unstimulated for 30 s and then treated with anti-BCR in the presence or absence of HD-m/lEVs, PRA-m/lEVs, or PRA-m/lEV-ICs for 210 s (indicated with an arrow). The mean of 5 HD is shown. In the inserted small figure, the linear slopes of calcium mobilization at 30 − 90-s period (linear) are shown. Data of five HD and median are shown. **B** The area under the curve of calcium mobilization in total B cells at 0 − 30-s (left panel), 30 − 120-s (center panel), and 120 − 240-s (right panel) periods. The data of 5 HD and median are shown. **C** The area under the curve of calcium mobilization in the 30 − 120-s (top panel) and 120 − 240-s (bottom panel) periods for transitional, CD27^−^IgM^+^, CD27^+^IgM^+^, CD27^+^IgM^−^, and CD27^−^IgM^−^ B cell subsets. Data of 5 HD and median are shown. **D** Representative histogram of tyrosine phosphorylation (p-Tyr, left panel) in B cells without stimulation or those treated with anti-IgM in the presence or absence of HD-m/lEVs, PRA-m/lEVs, or PRA-m/lEV-ICs. The mean fluorescence intensity of p-Tyr (right panel) in B cells of 7 HD donors and median are shown. **A–D** Kruskal − Wallis test with Dunn’s post-test. * *p* < 0.05, ** *p* < 0.01, *** *p* < 0.001, **** *p* < 0.0001

To determine whether the inhibition of B cell responses by m/lEVs could be detected early after BCR triggering, the mobilization of calcium and global phosphorylation of tyrosine were tested in B cells from HD (Fig. S3A). Decreased levels of calcium mobilization were suggested at 30 − 120 s of the kinetic in the presence of m/lEVs (Fig. 3A). A significantly lower slope of the curve was noted in the presence of PRA-m/lEV-ICs when compared to that in the absence of m/lEVs (Fig. 3A). However, the analysis of the area under the curve by time intervals revealed that the presence of m/lEVs did not significantly affect the levels of free calcium in total B cells when compared with that in the absence of vesicles (Fig. 3B). This discrepancy in the results could be based on the potential variations in calcium mobilization among different B cell subsets: transitional (CD19^+^CD24^hi^CD38^hi^), naive (CD19^+^CD38^low/−^IgM^+^CD27^−^), unswitched memory (CD19^+^CD38^low/−^IgM^+^CD27^+^), switched memory (CD19^+^CD38^low/−^IgM^−^CD27^+^), and CD27 negative memory (CD19^+^CD38^low/−^IgM^−^CD27^−^) (Fig. S3B). Further analysis of the data according to B cell subsets indicated a significant decrease in the levels of calcium mobilization in transitional (HD-m/lEVs and PRA-m/lEVs), unswitched memory (HD-m/lEVs and PRA-m/lEV-ICs), switched memory (HD-m/lEVs and PRA-m/lEV-ICs), and CD27 negative memory (HD-m/lEVs) B cells at 30 − 120 s of the kinetic in the presence of different m/lEVs (Fig. 3C). In addition, some significant changes were also detected at later time points (120–240 s) for switched memory (HD-m/lEVs and PRA-m/lEV-ICs) and double negative memory (HD-m/lEVs) B cells.

On the other hand and as expected, anti-BCR and pervanadate (positive control) stimulation led to increased expression of global phosphorylation of tyrosine (Fig. S3C). The presence of either of the m/lEVs pools that did not form ICs significantly reduced the phosphorylation after anti-BCR stimulus when compared with that in the absence of these vesicles (Fig. 3D).

These results further support m/lEVs suppress other events associated with the activation of B cells from HD, such as proliferation, tyrosine phosphorylation, and calcium mobilization. This phenomenon seems to occur as an early event of BCR signaling.

### Modulation of B cell activation by m/lEVs seems to be partially dependent on PS

To expand our understanding of how m/lEVs induce the modulation of B cell activation, some potential pathways were tested with a focus on the effect of these vesicles over B cell activation markers (or the reduction of CD69, CD80, and CD86 expression). m/lEVs may function as agonists of the inhibitory receptor FcγRIIb/CD32b because these vesicles can form ICs [13]. However, no significant differences were noted in the frequency of CD69^+^, CD80^+^, and CD86^+^ cells in anti-BCR stimulated B cells that were blocked with AT10 (a monoclonal antibody against CD32b) before their exposure to PRA-m/lEVs and PRA-m/lEV-ICs when compared with that in the absence of this blocking antibody (Fig. S4A-B). Moreover, as showed earlier, no differences were observed in the CD69, CD80, and CD86 expression of B cells between PRA-m/lEVs and PRA-m/lEV-ICs treatment (Fig. 2B,C). An alternative regulatory receptor of B cells that can be contacted by sialic acids of m/lEVs is CD22. Previously, sialic acids, such as α2,3- and α2,6-linked, have been reported in the surface of m/lEVs [36]. The treatment of m/lEVs with the lectins MAA or SNA that bind α2,3- and α2,6-linked sialic acids respectively, did not change the effect of these m/lEVs over B cell activation markers (Fig. S4C-D).

B cell interaction with m/lEVs could be also mediated by PS binding receptors [19, 37]. There was no difference in the PS content between m/lEVs from HD and patients with RA as measured through Annexin V staining (Fig. S4E-F). Annexin V has been used to block PS binding on EVs to target cells [38, 39]. The treatment of m/lEVs with Annexin V significantly reverted the effect of these vesicles over the CD69 and CD86 expression on B cells when compared with that after exposure of m/lEVs without Annexin V treatment (Fig. S4G). These data suggest that the inhibitory effect of m/lEVs on B cells is partially dependent on the PS binding receptors.

### m/lEVs also suppress B cell responses from patients with RA

**Fig. 4:**
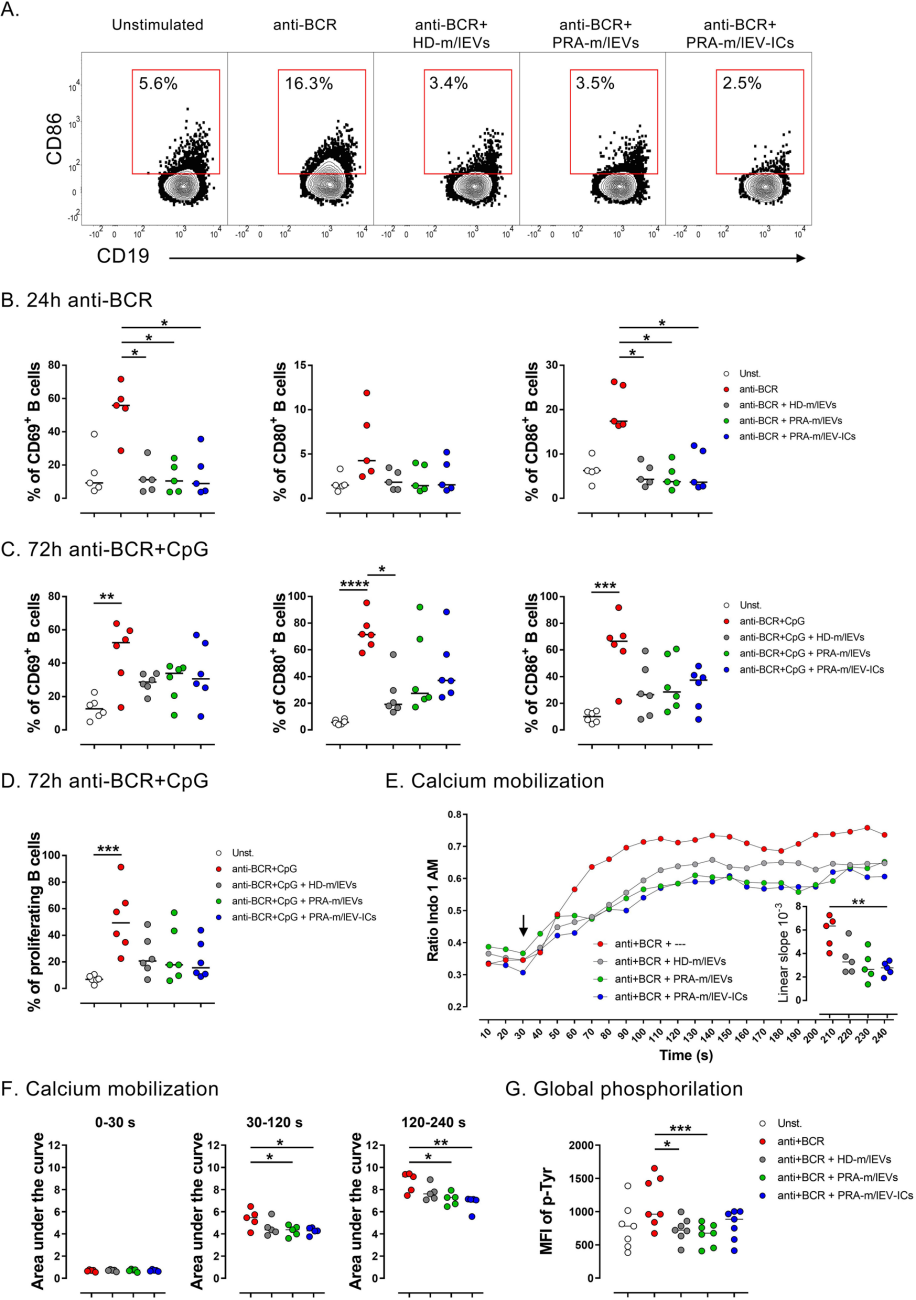
Regulatory effect of m/lEVs in B cells from patients with RA. **A** Representative contour plots of the CD86 expression in B cells from patients with RA, cultured for 24 h without stimulation (unstimulated, Unst) or with anti-BCR in the absence or presence of m/lEVs from HD (HD-m/lEVs), patients with RA (PRA-m/lEVs), and patients with RA-forming immune complexes (PRA-m/lEV-ICs). **B** The frequency of CD69^+^ (left panel), CD80^+^ (center panel), and CD86^+^ (right panel) B cells, cultured for 24 h without stimulation or with anti-BCR in the absence or presence of HD-m/lEVs, PRA-m/lEVs, or PRA-m/lEVs-ICs. Data of five patients with RA and median are shown. **C** The frequency of CD69^+^ (left panel), CD80^+^ (center panel), and CD86.^+^ (right panel) B cells, cultured for 72 h without stimulation or with anti-BCR and CpG in the absence or presence of HD-m/lEVs, PRA-m/lEVs, or PRA-m/lEVs-ICs. Data of six patients with RA and median are shown. **D** The frequency of proliferating B cells cultured of 72 h without stimulation or with anti-BCR and CpG in the absence or presence of HD-m/lEVs, PRA-m/lEVs, or PRA-m/lEVs-ICs. Data of six patients with RA and median are shown. **E** Kinetic of calcium mobilization of total B cells based on the ratio of Indo 1 AM bound/unbound in total B cells. After 30 s of acquisition, the B cells were stimulated with anti-BCR in the presence or absence of HD-m/lEVs, PRA-m/lEVs, or PRA-m/lEVs-ICs (indicated with an arrow). The mean of five patients with RA is shown. In the inserted small figure, the linear slopes of calcium mobilization in total B cells at 30 − 90-s period (linear) are shown. Data of five patients with RA and median are shown. **F** Area under the curve of calcium mobilization at the baseline (left panel), incremental (center panel), and post-incremental (right panel) phases of B cells. Data of five patients with RA and median are shown. **G** The median fluorescence intensity (MFI) of tyrosine phosphorylation (p-Tyr) in B cells without stimulation or with anti-IgM in the presence or absence of HD-m/lEVs, PRA-m/lEVs, or PRA-m/lEV-ICs. Data of five patients with RA and median are shown. **B–G** Kruskal − Wallis test with Dunn’s post-test. * *p* < 0.05, ** *p* < 0.01, *** *p* < 0.001, **** *p* < 0.0001

We proposed that the immunosuppressive effect of m/lEVs over B cell activation could be altered in autoimmune diseases, such as RA [40]. Accordingly, the effect of m/lEVs on B cells from patients with RA was evaluated following the analogous approach as for HD (Fig. 4A). Similarly, a decreased frequency of CD69^+^ and CD86^+^ B cells was observed after 24 h of anti-BCR treatment in the presence of m/lEVs when compared with the absence of these vesicles (Fig. 4B). However, although some tendency is evident, in most of these cases, no significant differences were observed in the frequency of CD69^+^, CD80^+^, and CD86^+^ B cells stimulated with anti-BCR and CpG regarding the presence of every m/lEV pool alone after both 24 h (Fig. S5A) and 72 h (Fig. 4C), but decreased proportions in CD69, CD80, and CD86 markers were detected when m/lEV pools were analyzed together at 72-h cultures (Fig. S5B). In addition, no significant changes in proliferation of B cells from RA patients were detected according to the type of m/lEVs (Fig. 4D); however, a significant decrease was observed when all m/lEV pools were analyzed together (Fig. S5B). Also, m/lEVs mediated a clearer down-modulation of CD80 and CD86 at 72 h than at 24 h (Fig. S5C). Comparable to B cells from HD, the tendency to lower the production of IL-6, IL-10, and TNF-α was detected with m/lEVs, which showed no significance in outcome for B cells from patients with RA cultured for 24 and 72 h (Fig. S5D-E).

Calcium mobilization and global phosphorylation of tyrosine of anti-BCR stimulated B cells from patients with RA were evaluated. As showed for HD, reduced calcium mobilization was detected in B cells of patients in the presence of m/lEVs (Fig. 4E), and a reduced slope of the curve was evident in the presence of PRA-m/lEV-ICs when compared to that in the absence of the vesicles (Fig. 4E). Particularly, the effect of the m/lEVs was observed at 30 − 120 s and 120 − 240 s, which was statistically significant only for PRA-m/lEVs and PRA-m/lEV-ICs (Fig. 4F). The following significant differences were observed in B cell subsets with three different sources of vesicles: HD-m/lEVs (for CD27^+^IgM^−^ and CD27^−^IgM^−^), PRA-m/lEVs (for CD27^−^IgM^+^ and CD27^+^IgM^+^), and PRA-m/lEV-ICs (for all evaluated subsets) (Fig. S5F). Similarly, lower global phosphorylation of tyrosine in anti-BCR stimulated B cells from patients with RA was recorded in the presence of PRA-m/lEVs, but not with the other vesicles (Fig. 4G).

As observed with B cells from HD, m/lEVs seem to be able to inhibit several responses of B cells from patients with RA.

### T cell-dependent activation of B cells exposed to m/lEVs increase the production of IgM and IgG but do not affect the generation of plasmablast cells

**Fig. 5:**
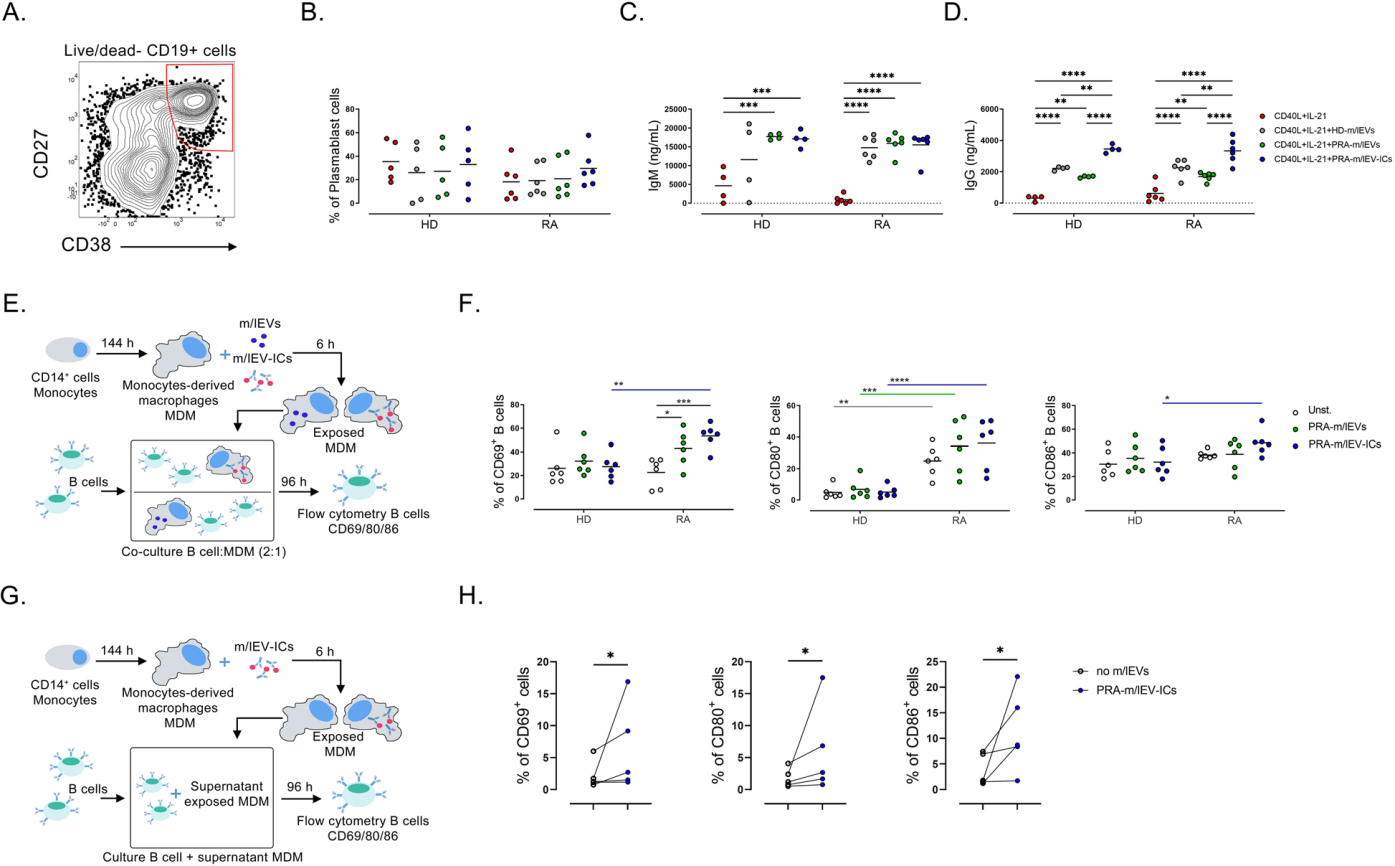
m/lEVs can directly increase B cell production of antibodies and indirectly activate autologous B cells from patients with RA by innate immune cells. **A** Representative contour plot of plasmablast population CD27^+^CD38^hi^ in enriched B cells cultured with CD40L and IL-21 for 7 days. Plasmablasts were analyzed inside of lymphocyte, live/dead negative, and CD19 + regions. **B** Frequency of plasmablast cells as explained in **A**, in the absence or presence of HD-m/lEVs, PRA-m/lEVs, or PRA-m/lEVs-ICs. Data of five HD, six patients with RA, and median are shown. **C**, **D** Levels of **C** IgM and **D** IgG in supernatant of culture of B cells as explained in **A**, in the absence or presence of HD-m/lEVs, PRA-m/lEVs, or PRA-m/lEVs-ICs. Line indicates lowest detection limit of the ELISA (2 ng/mL). **E** Monocyte-derived macrophages (MDM) from HD and patients with RA were exposed or not to PRA-m/lEVs or PRA-m/lEVs-ICs for 6 h. Autologous B cells were added to previously washed MDM (B cells: MDM ratio of 1-2:1) and incubated for 96 h. **F** Frequency of CD69^+^, CD80^+^, and CD86.^+^ B cells from cocultures with MDM unstimulated (Unst) or treated with PRA-m/lEVs or PRA-m/lEV-ICs, as detailed in E. **G** MDM from patients with RA were exposed or not to PRA-m/lEV-ICs for 6 h. Autologous B cells were cultured with the supernatant of exposed MDM and incubated for 96 h. **H** Frequency of CD69+, CD80+, and CD86+ B cells cultured with the supernatant of MDM unstimulated (Unst) or treated with PRA-m/lEV-ICs, as detailed in G. Data of six HD and six patients with RA are shown. **B–D**, **F** Two-way ANOVA test with Šidák post-test. **H** Wilcoxon test. * *p* < 0.05, ** *p* < 0.01, *** *p* < 0.001, **** *p* < 0.0001

To evaluate the effect of m/lEVs over the generation of plasmablast cells and production of antibodies, enriched B cells from HC and patients were cultured in complete media with CD40L and IL-21 for 7 days in the absence or presence of m/lEVs. Plasmablast cells were evaluated in live CD19 + cells according to CD27 and CD38 expression (Fig. 5A). No differences in frequency of plasmablasts were observed between the presence or absence of m/lEVs (Fig. 5B). However, increased concentration of IgM (Fig. 5C) and IgG (Fig. 5D) were found in supernatants of cultures with the presence of PRA-m/lEVs and PRA-m/lEV-ICs for B cells of HC, and with all m/lEVs for B cells of patients compared to the absence of m/lEVs. These data suggest that despite m/lEVs have a down modulatory effect on early B cell activation through the BCR, they seem also to promote the production of antibodies in a context that resembles T cell cooperation.

### MDM exposed to PRA-m/lEVs and PRA-m/lEV-ICs promote the activation of B cells from patients with RA

The finding that m/lEVs reduce B cell activation was unexpected, particularly for patients with autoimmune diseases, such as RA, which involved elevated concentrations of circulating m/lEVs and autoreactive B cells [11, 12, 40]. Considering these results and the fact that B cells are activated in lymphoid follicles, a restricted anatomical location, we propose that the effect of circulating m/lEVs on B cells could be indirect. The indirect effect may be achieved through antigen-presenting cells (APCs) that can deliver antigens to B cells in the secondary lymphoid organs [41, 42]. In addition, as previously described, macrophages differentiated in presence of PRA-m/lEV-ICs can produce high levels of IL-1β, IL-6, and TNF-α [42], which could influence the behavior of B cells [43].

To test this potential pathway, MDM previously exposed to PRA-m/lEVs or PRA-m/lEV-ICs were cocultured with autologous B cells (Fig. 5E). We observed a higher frequency of CD69^+^ and CD86^+^ B cells from RA patients after their coculture with MDM exposed to PRA-m/lEV-ICs when compared with B cells from HD (Fig. 5F). Moreover, an increased frequency of CD80^+^ B cells was noted in all cocultures from patients when compared with that from HD, irrespective of the presence of m/lEVs (Fig. 5F). Interestingly, an expanded frequency of CD69^+^ B cells from patients with RA was observed when these cells were cocultured with MDM exposed to PRA-m/lEVs and PRA-m/lEV-ICs than when cocultured with untreated MDM. This effect was not observed in B cells from HD (Fig. 5F).

Supernatant of MDM monocultures exposed to PRA-m/lEVs and PRA-m/lEV-ICs showed higher levels of IL-1β, IL-6, IL-8, IL-10, and TNF-α than in the absence of these vesicles (Fig. S6A), suggesting a role of soluble components on B cell activation in cocultures. To further evaluate this hypothesis, enriched B cells from patients with RA were cultured with supernatant of MDM previously exposed or not to PRA-EV-ICs (Fig. 5G). Increased frequency of CD69^+^, CD80^+^, and CD86^+^ B cells were observed in cultures with supernatants of MDM exposed to m/lEV-ICs compared with the absence of these structures (Fig. 5H). These data suggest that m/lEV-ICs-exposed MDM induce the activation of B cells in patients with RA, partially through soluble mediators.

## Discussion

In the present study, we characterized the effect of m/lEVs and m/lEV-ICs from patients with RA in B cell activation. We found that these EVs reduced the expression of activation markers, decreased calcium mobilization, and lowered tyrosine phosphorylation of in vitro-activated B cells. This immunosuppressive effect of m/lEVs was observed either in B cells from HD or in patients with RA activated through BCR alone (signal 1), or with a TLR-9 agonist (CpG) that decreases the threshold of B cell activation [44] (signals 1 and 2). The suppressive effect of m/lEVs was not noted in long cultures when B cells were activated with molecules that resemble T cell cooperation, either for B cells from HD or patients with RA. Furthermore, B cells from patients were more prone to undergo activation after coculture with m/lEVs-exposed MDM than B cells from HD. These results suggest that m/lEVs play a modulatory role over B cell activation that is potentially altered in patients with RA.

### Potential modulation mechanisms of B cell responses by m/lEVs

A possible explanation for the direct inhibition mediated by m/lEVs over B cell responses could be that m/lEVs mask the stimulation by competing for B cell agonists. However, we discarded this hypothesis because no significant changes in B cell activation were noted when m/lEVs were added before or after the BCR and CpG stimulation. Hence, it is worth proposing that m/lEVs can activate some regulatory pathways in B cells. Previously, our research group had reported that B cells can interact with and internalize m/lEVs [20], and therefore the regulatory effect of m/lEVs could be due to the recognition of surface or endosomal receptors present on the B cells. In support of this idea, it has been reported that exosomes, other m/lEVs of smaller sizes (30 − 100 nm) than m/lEVs, decreased the phospho-Btk expression in anti-BCR stimulated B cells from HD [45]. Btk phosphorylation is a critical step in B cell activation upon BCR ligation that is mainly regulated by phosphatases SHP1 (downstream of the CD22 pathway) and SHIP1 (recruited after FcγRIIb ligation), among other molecules [46]. Reduced calcium mobilization and global phosphorylation in B cells have been described upon FcγRIIb and CD22 ligation [47]. These receptors contain immunoreceptor tyrosine-based inhibition motif domains that activate the phosphatases mentioned earlier [47]. However, we noted no significant effect of blocking FcγRIIb and CD22 ligands regarding the B cell responses to m/lEVs and m/lEV-ICs.

An alternative pathway could be the binding of PS on m/lEVs. Previously, it has been described that the uptake of m/lEVs by activated platelets [38] and endothelial cells [39] is dependent on PS binding. We observed that Annexin V can partially reverse the regulatory effect of m/lEVs over B cells. Normally, the removal of apoptotic bodies by PS binding results in regulatory effects that have been demonstrated mainly in other APCs, such as macrophages [48]. It is possible that similar signaling could also occur in B cells through PS binding receptors, such as CD300, as previously described for B cells [49] or other receptors, such as TIM1 and TIM4, also described in B cells that can recognize PS and regulate the B cell responses in mice [50–52]. Thus, further studies are required for a better understanding of the interactions of m/lEVs with B cells and to yield the suppressive pathways induced by these m/lEVs. Furthermore, it is worth mentioning that naturally occurring immunosuppressive m/lEVs have been previously described in other models, for instance, those derived from immunosuppressive APCs [53]. However, the vesicles from RA patients are not inhibitory on their own as they can classically activate mononuclear phagocytes [42].

Checkpoint molecules that are capable of downregulating immune responses, such as PD-1 and CTLA-4 [54], are other potential candidates that explain the suppression induced by m/lEVs in B cell early responses. It has been reported that exosomes increased the PD-1 expression (a negative regulator of activation) in CD40L plus IL-4 stimulated B cells when compared with that in the absence of these m/lEVs [45]. PD-L1 (a ligand of PD-1) is also expressed on B cells upon BCR stimulation [55]. Therefore, it is worth proposing that the homotypic interaction of activated B cells through PD-L1^+^ and PD-1 may further contribute to the downregulation of these cells after treatment with anti-BCR and m/lEVs. Interestingly, patients with RA showed a lower frequency of PD-L1^+^ B cells in vitro when compared to HD, although the expression of this molecule increased after successful treatment [56]. Thus, B cells from patients with RA may be less responsive to m/lEVs due to the dysregulation of immune checkpoint molecules. Further studies are warranted to unveil the participation of these molecules and other receptors, such as CTLA-4, CD72, CD95, and CD5 in the regulation of B cell activation induced by m/lEVs.

### Evidence of modulation of B cells by m/lEVs

We observed that different sources of m/lEVs decrease the expression of activation markers and the proliferation of B cells. Previously, increased CD69 expression and proliferation of B cells in PHA-stimulated PBMC exposed to m/lEVs has been reported when compared with that in the absence of these structures [57]. Differences between these past results and the present results can be explained by the type of cells used for culturing. The effect of m/lEVs in promoting the activation, rather than the inhibition, of B cells can be an indirect response because monocytes, which are present in the PBMCs, are the most efficient leukocytes to bind with and then internalize these vesicles [20]. Monocytes can produce high levels of proinflammatory cytokines (i.e., IL-1β, TNF-α, and IFN-α) after the treatment with m/lEVs and mainly when they form ICs [19, 27]. In support of the suppressive effect of m/lEVs in B cell proliferation, it was recently suggested that exosomes isolated from HD and patients with head and neck squamous cell carcinoma inhibited B cell proliferation from HD [45].

The regulatory effect of m/lEVs was observed since the early time points of B cell activation through the BCR alone or together with TLR-9 in HD and patients with RA. For example, we observed a reduced slope of the curve in the presence of m/lEV-ICs, which lead to a delay in reaching the maximum calcium signal of B cells from HD in the presence of vesicles; for patients with RA, the presence of m/lEVs avoid B cells to reach the maximal signal (as observed in the absence of m/lEVs). Although it remains unclear why these particular responses of B cells from patients with RA to m/lEVs happen, this phenomenon can be explained through a potential scenario wherein B cells from patients with RA have been chronically stimulated in vivo, leading to a post-activated/exhausted state of B cell, which has been associated with hyporesponsive to B cell stimulation [58, 59].

Increased levels of IgM and IgG in the presence of m/lEVs were detected. Previous work described a reduced proliferation of B cells with increased antibody titers in mice after the treatment with ovalbumin and synthetic sialoside molecules that bind CD22 with high affinity [60]. These results are similar to ours, suggesting that the effect of m/lEVs could be mediated by CD22 ligands. Sialic acids are known CD22 ligands previously reported on the surface of EVs [36]. However, blocking these sialic acids with some lectins (α2,3- and α2,6-linked sialic acids) did not change the effect of m/lEVs over B cells. We cannot rule out that other ligands of CD22 or Siglec receptor (also described in B cells [61]) could be mediating this response. Irrespective of a potential role of these molecules, the data suggest that although m/lEVs have a suppressive role at early responses mediated by the BCR and some coreceptors, in later responses when the presence of signals provided by T follicular helper (Tfh) cells (costimulatory molecules and cytokines) are required, a contrasting role of m/lEVs is observed. This could be particularly important for patients with seropositive RA because an excess of these m/lEVs together with B cell infiltration has been reported in inflamed joints [11, 62]. Therefore, the dual role of these m/lEVs in B cell function needs to be carefully considered if these vesicles want to be handled as therapeutic options in the future.

### Indirect effect of m/lEVs on B cell responses through innate immune cells

As shown in this study, m/lEVs directly suppress the activation of enriched B cells stimulated through the BCR. However, the presence of other cells (such as monocytes and macrophages) previously exposed to m/lEVs could modify this outcome. Earlier, our group reported that monocytes exposed to m/lEVs (derived from platelets forming ICs) increased their CD69 and CD64 expression and the production of IL-1β, TNF-α, and IFN-α [19, 27]. Moreover, MDM differentiated in the presence of PRA-m/lEVs-ICs showed an increased production of TNF-α, IL-1β, and IL-6 when compared with that in the absence of these vesicles [42]. Because of the proinflammatory effect of m/lEVs over innate cells and the role of macrophages in the capture of antigens and noncanonical presentation to B cells in the lymph tissues [63], we evaluated the effect of PRA-m/lEVs- and PRA-m/lEV-ICs-exposed MDM on B cell activation. In the lymph nodes, small antigens (12 − 14 kDa) can be directly distributed to B cells, but large (approximately 240 kDa) and complex antigens (including complete virus, bacteria, and potentially m/lEVs because of their size and composition) are captured by APCs and then delivered to B cells [64]. We confirm that PRA-m/lEVs- and PRA-m/lEV-ICs-treated MDM produce elevated levels of proinflammatory cytokines and induced higher frequency of autologous CD69^+^ B cells in patients with RA compared to HD. Although it has been shown that direct contact between B cells and macrophages is required for antigen transfer [65], suggesting that a noncanonical presentation by innate cells can be central in B cell activation by m/lEV-IC, we observed that the soluble factors produced by MDM can promote the expression of activation markers in B cells without the presence of cell contact. Thus, these data suggest macrophages previously exposed to PRA-m/lEVs and PRA-m/lEV-ICs could promote B cell activation in patients with RA, at least in part, because of the secretion of soluble molecules such as cytokines.

### Limitations of the study

Regarding the limitations, it is important to mention that purified IgG from patients was used to produce PRA-m/lEV-ICs; this IgG could bias the measurement of this immunoglobulin in the supernatants of B cell cultures, as suggested by higher IgG levels (but not IgM) in cultures in which such opsonized vesicles were used. In addition, m/lEVs could have differential effects on B cells in longer time periods and/or in presence of other stimulants (like CD40L and IL-21), as suggested by the increased antibody production observed after 7 days of stimulation. Further investigation is required for a better understanding of this phenomenon. Lastly, it was not possible to evaluate the cocultures in presence of HD-m/lEVs due to the limited availability of cells from patients; then, we could not discern the specific contribution of the vesicle sources in the observed upregulation of activation markers.

## Conclusions

Our data suggest that circulating m/lEVs act as potential promoters of B cell tolerance because they prevent the activation of these cells through the BCR. This finding should be associated with the fact that naive B cells are activated in lymphoid follicles rather than in circulation. The known proinflammatory effect of m/lEVs and m/lEV-ICs from patients with RA over innate immune cells, such as macrophages and monocytes, leads to increased production of soluble factors (such as IL-6, IL-10, TNF-α, and IL-1β) [42] and a potential noncanonical presentation of autoantigens. Therefore, it is possible to propose that, in RA, and maybe in other autoimmune and inflammatory diseases, the effect of m/lEVs over B cells is altered because of the proinflammatory impact of these m/lEVs, especially those forming ICs, over the innate immune cells. Interestingly, CD16^+^ monocytes are expanded in circulation and synovial fluid of patients with RA [13, 66]. We observed CD16^+^, but not CD16^−^, monocytes exposed to PRA-m/lEV-ICs secreted increased levels of IL-1β and TNF-α (Fig. S6B). These cells can improve B cell activation in lymph nodes in a macaque model of dengue virus infection, in part mediated by cytokine production [67], which could suggest a similar scenario in RA with the reported increase in CD16^+^ monocytes and effect of PRA-m/lEV-ICs exposure. The immune microenvironment is key in the regulation of B cell response [43], suggesting mononuclear phagocytes contacted by m/lEVs could promote the loss of tolerance by fostering chronic and uncontrolled B cell activation. In addition, the increased antibody production suggests m/lEVs could have agonist effects in later B cell responses associated with T cell coestimulation. Therefore, blocking the effect of m/lEVs over innate cells, such as CD16^+^ monocytes or macrophages, could be a promising target for immunotherapy in RA.

## Supplementary Information

**Additional file 1: Table S1.** List of antibodies. **Figure S1.** Effect of m/lEVs on the induction of activation markers in B cells from HD. **Figure S2.** Impact of EVs on cytokine levels of B cells from HD. **Figure S3.** Calcium mobilization of B cells from HD. **Figure S4.** The regulatory effect of m/lEVs on B cell responses seems to be partially dependent on phosphatidylserine. **Figure S5.** Effect of m/lEVs on the induction of activation markers, cytokine levels and calcium mobilization in B cells from patients with RA. **Figure S6.** Effect of m/lEVs on cytokine production by macrophages and subsets of monocytes.

## Data Availability

All data generated during this study are available from the corresponding author on reasonable request.

## Abbreviations

ANOVA: Analysis of variance
APCs: Antigen-presenting cells
BAFF: B cell activator factor
BCR: B cell receptor
BSA: Bovine serum albumin
CCP: Cyclic citrullinated peptide
CD: Cluster of differentiation
CpG: Short DNA island containing enriched motive of unmethylated 5′-C-phosphate-G-3′ sequence
CTLA-4: Cytotoxic T-lymphocyte Antigen 4
DAS28: Disease activity score 28
DNA: Deoxyribonucleic acid
EDTA: Ethylenediaminetetraacetic acid
EVs: Extracellular vesicles
FACS: Fluorescence-activated cell sorter
FcγRIIb: Fc gamma receptor IIb
FSC-A: Forward scatter—area
HD: Healthy donor
ICAM: Intercellular adhesion molecule
ICs: Immune complexes
iFBS: Inactivated fetal bovine serum
Ig: Immunoglobulin
IL: Interleukin
MAA: *Maackia amurensis* lectin agglutinin
MDM: Monocyte-derived macrophages
miRNA: MicroRNA
m/lEVs: Medium/large extracellular vesicles
m/lEV-ICs: M/lEVs forming immune complexes
PBMCs: Peripheral blood mononuclear cells
PBS: Phosphate-buffered saline
PD-1: Programmed death-1
PHA: Phytohaemagglutinin
PRA: Patient with rheumatoid arthritis
PS: Phosphatidylserine
RA: Rheumatoid arthritis
RNA: Ribonucleic acid
RT: Room temperature
SDE: Serum depleted of m/lEVs
SHP1: Src homology 2 domain phosphatase-1
SHIP1: SH2 domain-containing inositol-5'-phosphatase-1
SNA: *Sambucus nigra* lectin agglutinin I
SSC-A: Side scatter—area
TIM: T cell/transmembrane, immunoglobulin, and mucin
TLRs: Toll-like receptors
TNF: Tumor necrosis factor
