# Supplementary material for: Modulation of B cell activation by extracellular vesicles and potential alteration of this pathway in patients with rheumatoid arthritis
Source: Arthritis Res Ther. 2022 Jul 16;24:169. doi: 10.1186/s13075-022-02837-3 (PMC9287863; doi:10.1186/s13075-022-02837-3)
Supplement: Supplementary file 1 — Additional file 1: Table S1. List of antibodies. Figure S1. Effect of m/lEVs on the induction of activation markers in B cells from HD. Figure S2. Impact of EVs on cytokine levels of B cells from HD. Figure S3. Calcium mobilization of B cells from HD. Figure S4. The regulatory effect of m/lEVs on B cell responses seems to be partially dependent on phosphatidylserine. Figure S5. Effect of m/lEVs on the induction of activation markers, cytokine levels and calcium mobilization in B cells from patients with RA. Figure S6. Effect of m/lEVs on cytokine production by macrophages and subsets of monocytes. [file 13075_2022_2837_MOESM1_ESM.docx]

**SUPPLEMENTARY INFORMATION**

**1. Supplementary Tables.**

**Table S1. List of antibodies.**

| Antibody | Fluorochrome | Isotype | Clone | Company | Panel |
| --- | --- | --- | --- | --- | --- |
| CD14 | RD1 | Mouse IgG2b | 322A-1 | Beckman Coulter | Monocyte culture |
| CD16 | FITC | Mouse IgG1, κ | 3G8 | BD | Monocyte culture |
| CD19 | V650 | Mouse IgG1, κ | HIB19 | Biolegend | B cell activation/Calcium Mobilization/ Global phosphorylation/Co-culture |
| CD21 | FITC | Mouse IgG1, κ | Bu32 | Biolegend | B cell activation/Calcium Mobilization |
| CD24 | V605 | Mouse IgG2a, κ | ML5 | Biolegend | B cell activation/Calcium Mobilization |
| CD27 | PE-Cy7 | Mouse IgG1, κ | O323 | Biolegend | B cell activation/Calcium Mobilization |
| CD38 | V785 | Mouse IgG1, κ | HIT2 | Biolegend | B cell activation/Calcium Mobilization/ Global phosphorylation |
| CD41a | PE | Mouse IgG1, κ | HIP8 | Biolegend | EV characterization |
| CD69 | V711 | Mouse IgG1, κ | FN50 | Biolegend | B cell activation/Calcium Mobilization/Co-culture |
| CD80 | PE | Mouse IgG1, κ | 2D10 | Biolegend | B cell activation/Calcium Mobilization/Co-culture |
| CD86 | V510 | Mouse IgG2b, κ | IT2.2 | Biolegend | B cell activation/Calcium Mobilization/Co-culture |
| IgD | A700 | Mouse IgG2a, κ | IA6-2 | Biolegend | B cell activation |
| IgM | PE-CF594 | Mouse IgG1, κ | MHM-88 | Biolegend | B cell activation/Calcium Mobilization |
| phospho-Tyrosine | eFluor450 | Mouse IgG2b | PY20 | eBiosciences | Global phosphorylation |
| F(ab’)2 anti-IgG | AF488 | Goat F(ab')₂ Fragment | Polyclonal | Jackson Immunoresearch | EV characterization |

**2. Supplementary Figures.**


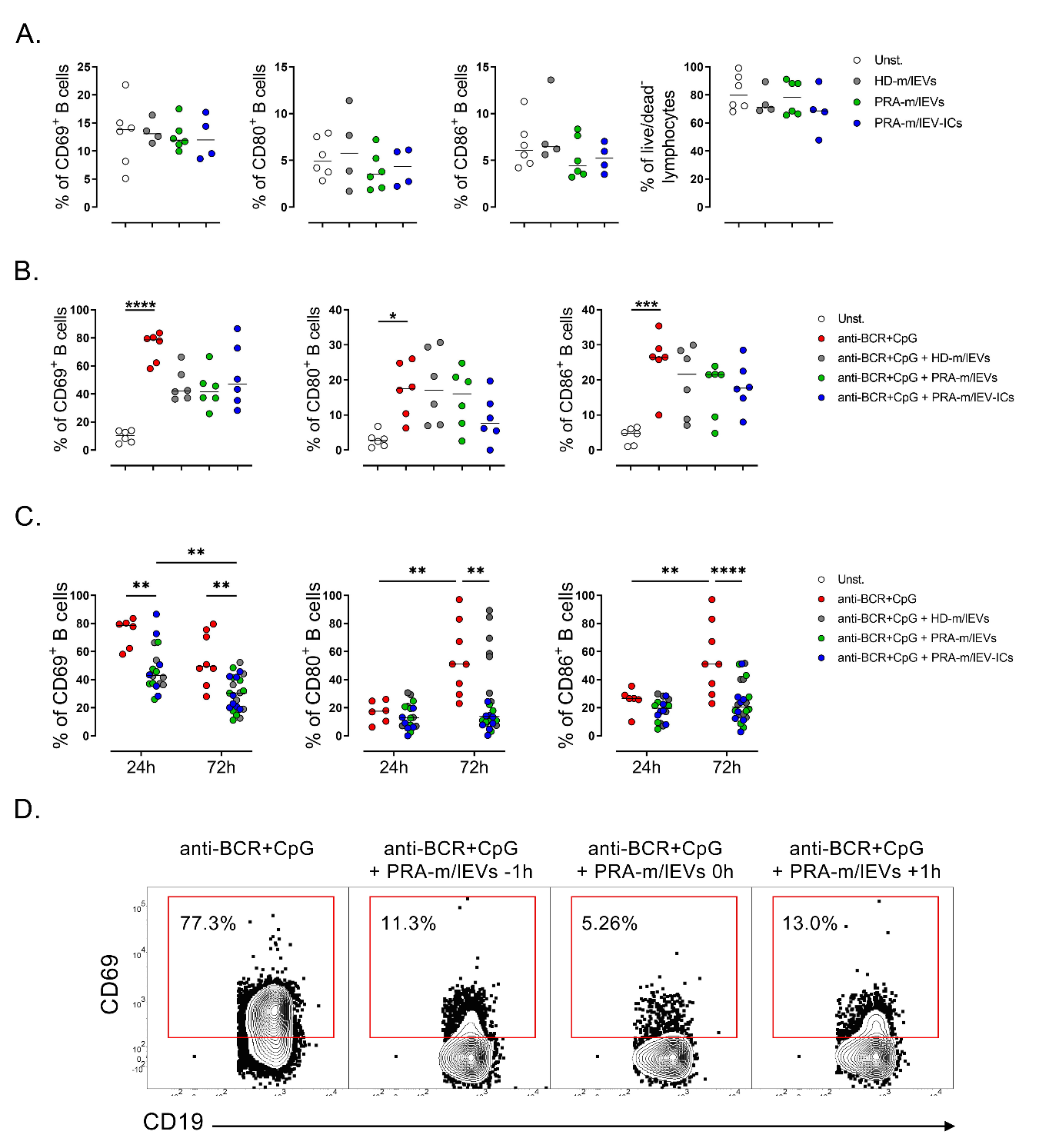


**Figure S1.** **Effect of m/lEVs on the induction of activation markers in B cells from HD.** **A.** Frequency of CD69^+^, CD80^+^, CD86^+^, and live/dead^−^ B cells, cultured for 24 h without stimulation or in presence of HD-m/lEVs, PRA-m/lEVs, or PRA-m/lEV-ICs. Data of five HD donors and median are shown. **B.** Frequency of CD69^+^ (left), CD80^+^ (center), and CD86^+^ (right) B cells, cultured for 24 h without stimulation or with anti-BCR and CpG in absence or presence of HD-m/lEVs, PRA-m/lEVs, or PRA-m/lEV-ICs. Data of six HD donors and median are shown. **C.** Frequency of CD69^+^ (left), CD80^+^ (center), and CD86^+^ (right) B cells, cultured for 24 h and 72 h in presence or not of HD-m/lEVs, PRA-m/lEVs, or PRA-m/lEV-ICs (all vesicles combined). Data of 24 h culture from 6 HD and 72 h from 8 HD. **D.** Representative contour plots of CD69 expression in B cells from HD cultured for 72 h with anti-BCR and CpG in absence or presence of EVs from patients with RA (PRA-m/lEVs) one hour before, simultaneously, or one hour after anti-BCR and CpG stimulation. One representative of three independent experiments. **A-B.** Kruskal−Wallis test with Dunn’s post-test. **C.** Two-way ANOVA test with Šidák post-test. * p<0.05, ** p<0.01, *** p<0.001, **** p<0.0001.


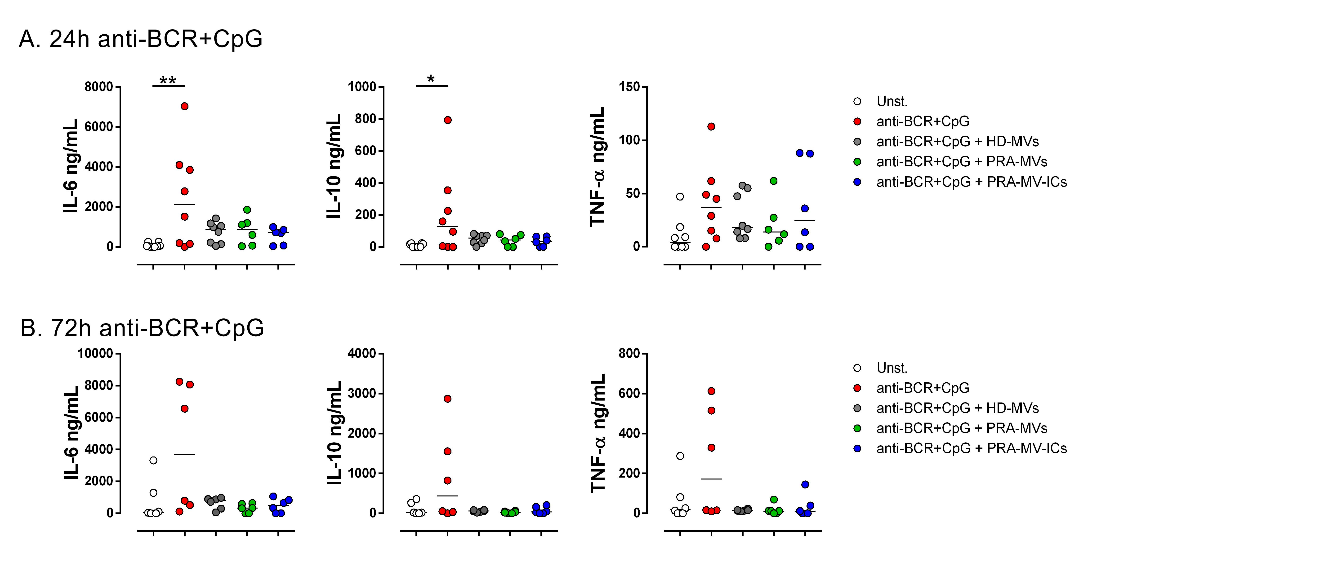


**Figure S2. Impact of EVs on cytokine levels of B cells from HD.** **A-B.** Levels of IL-6 (left), IL-10 (center), and TNF-α (right) in the supernatant of B cells cultured for 24 h (**A.**) and 72 h (**B.**) without stimulation or with anti-BCR and CpG in the absence or presence of HD-m/lEVs, PRA-m/lEVs or PRA-m/lEV-ICs. Data of six to eight HD donors and median are shown. **A-B.** Kruskal−Wallis test with Dunn’s post-test. * p<0.05, ** p<0.01.

**
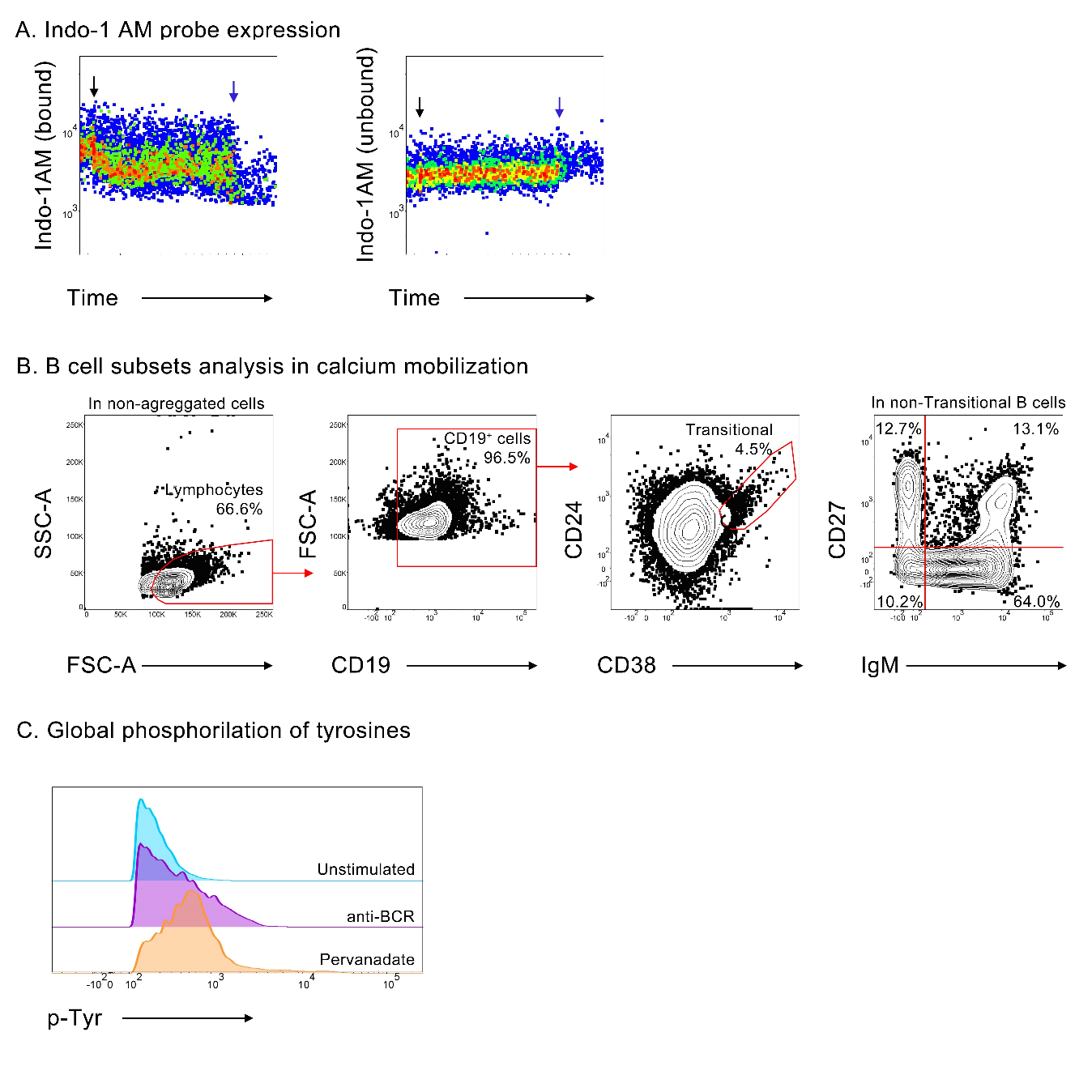
**

**Figure S3. Calcium mobilization of B cells from HD.** **A.** Representative pseudocolor plot showing the expression of Indo-1 AM bound (left) and unbound (right) to calcium in B cells from HD. B cells were kept unstimulated for 30 s and then treated with anti-BCR in the absence or presence of EVs (black arrow) for 210 s. Latter, as positive control, cells were stimulated with Ionomycin for 120 s (blue arrow). **B.** Strategy of analysis for B cell subsets used for calcium mobilization. Representative contour plots of lymphocytes, selected by SSC-A and FSA-A parameters, B cells defined as CD19^+^ cells and B cell subsets: transitional (CD19^+^CD24^hi^CD38^hi^), naive (CD19^+^CD38^low/−^IgM^+^CD27^−^), unswitched memory (CD19^+^CD38^low/−^IgM^+^CD27^+^), switched memory (CD19^+^CD38^low/−^IgM^−^CD27^+^), and CD27 negative memory (CD19^+^CD38^low/−^IgM^−^CD27^−^) subsets. **C.** Representative histogram of tyrosine phosphorylation (p-Tyr) of enriched B cells from HD without stimulation (blue), with anti-BCR stimulation (purple) or treated with pervanadate (orange, positive control).


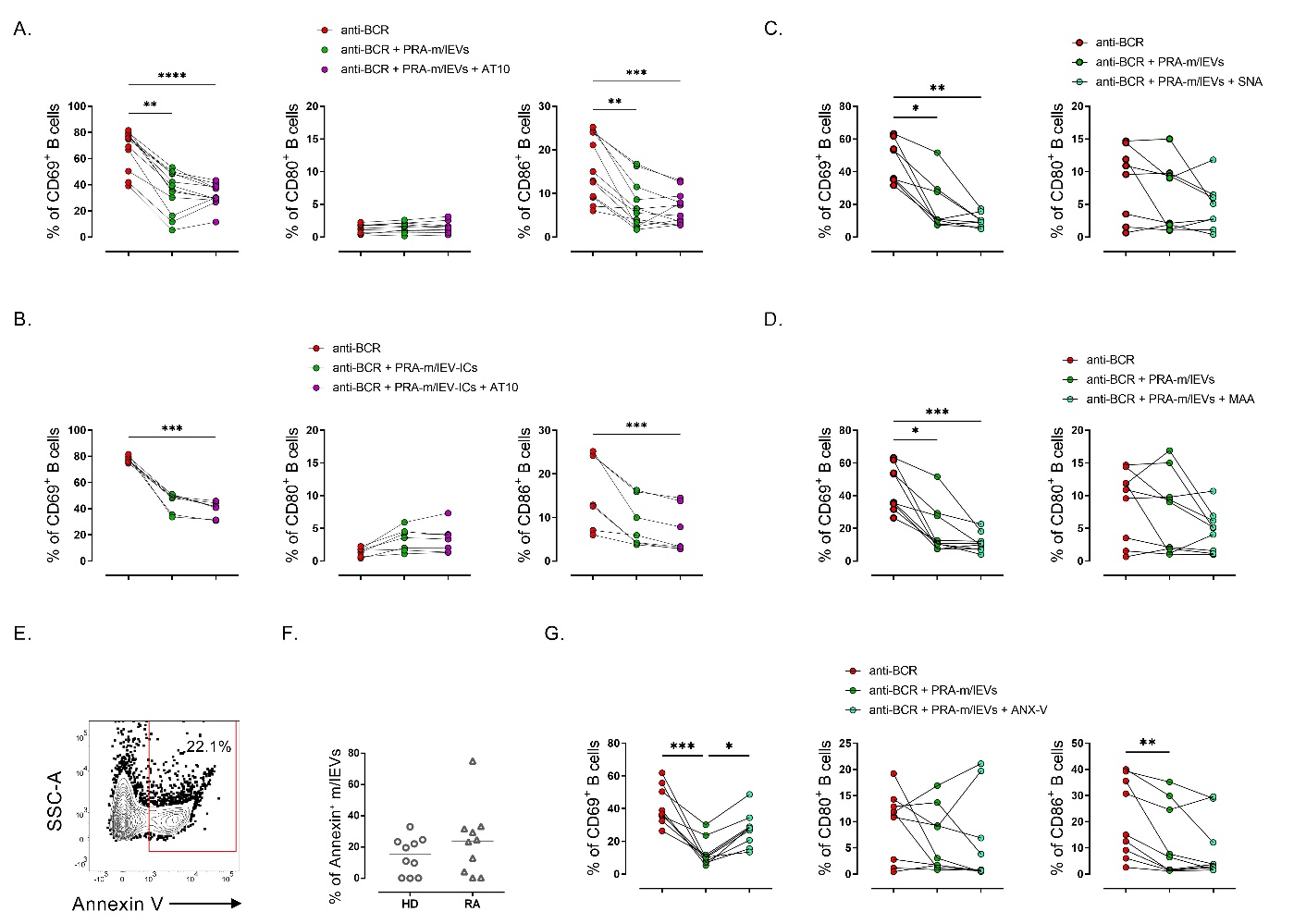


**Figure S4.** **The regulatory effect of m/lEVs on B cell responses seems to be partially dependent on phosphatidylserine.** **A-B.** Enriched B cells from HD were pre-incubated or not with AT10 (anti-FcγRIIb) and stimulated with anti-BCR together with **A.** m/lEVs (PRA-m/lEVs, n = 12) or **B.** m/lEVs forming immune complexes (PRA-m/lEV-ICs, n = 7) from patients with RA. Data of CD69^+^, CD80^+^, and CD86^+^ B cells and median are shown. **C-D.** Enriched B cells were stimulated with anti-BCR and PRA-m/lEVs earlier incubated with or without lectins, **C.** SNA (from *Sambucus Nigra,* n = 8) or **D.** MAA (from *Maackia amurensis,* n = 9). Data of CD69^+^ and CD80^+^ B cells and median are shown. **E.** Representative Annexin V staining of circulating m/lEVs from an HD. **F.** Frequency of Annexin V^+^ m/lEVs from HD and patients with RA. Data of EVs from ten HD and ten patients with RA and median are shown. Mann−Whitney test. **G.** Enriched B cells were treated with anti-BCR and PRA-m/lEVs previously incubated with or without Annexin V. Data of CD69^+^, CD80^+^, and CD86^+^ B cells and median are shown (n = 9). **A-D, G.** Friedman test with Dunn post-test. * p<0.05, ** p<0.01, *** p<0.001, **** p<0.0001.


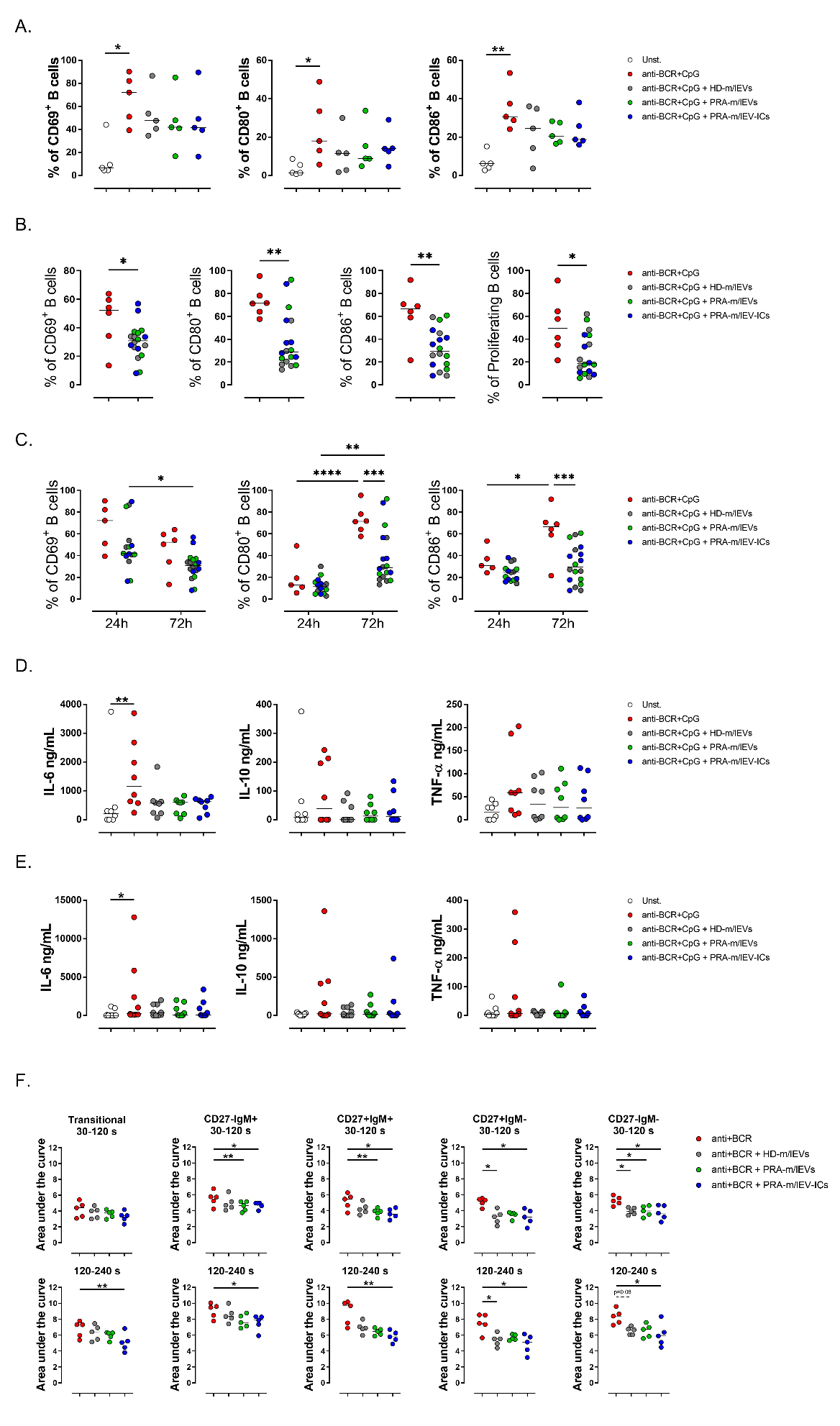


**Figure S5.** **Effect of m/lEVs on the induction of activation markers, cytokine levels and calcium mobilization in B cells from patients with RA.** **A.** Frequency of CD69^+^ (left), CD80^+^ (center) and CD86^+^ (right) B cells, cultured for 24 h without stimulation or with anti-BCR and CpG in absence or presence of HD-m/lEVs, PRA-m/lEVs or PRA-m/lEV-ICs. Data of five patients with RA and median are shown. **B.** Frequency of CD69^+^ (left), CD80^+^ (center) and CD86^+^ (right) B cells, cultured for 72 h with anti-BCR and CpG in absence or presence of m/lEVs. Data of six patients with RA and median are shown. **C.** Frequency of CD69^+^ (left), CD80^+^ (center), and CD86^+^ (right) B cells, cultured for 24 h and 72 h in presence or not of HD-m/lEVs, PRA-m/lEVs, or PRA-m/lEV-ICs (all vesicles combined). Data of 24 h culture from five and 72 h from six patients with RA. **D-E.** Levels of IL-6 (left), IL-10 (center), and TNF-α (right) in the supernatant of B cells cultured for 24 h (**D.**) and 72 h (**E.**) without stimulation or with anti-BCR and CpG in the absence or presence of HD-m/lEVs, PRA-m/lEVs or PRA-m/lEV-ICs. Data of eight to nine patients with RA and medians are shown. **F.** Area under the curve of calcium mobilization at 30-120 s (top) and 120-240 s (bottom) periods for transitional, CD27^−^IgM^+^, CD27^+^IgM^+^, CD27^+^IgM^−^, and CD27^−^IgM^−^ B cell subsets. Data of five patients with RA and median are shown. **A, D-F.** Kruskal−Wallis test with Dunn’s post-test. **B.** Mann−Whitney test. **C.** Two-way ANOVA test with Šidák post-test. * p<0.05, ** p<0.01, *** p<0.001, **** p<0.0001.


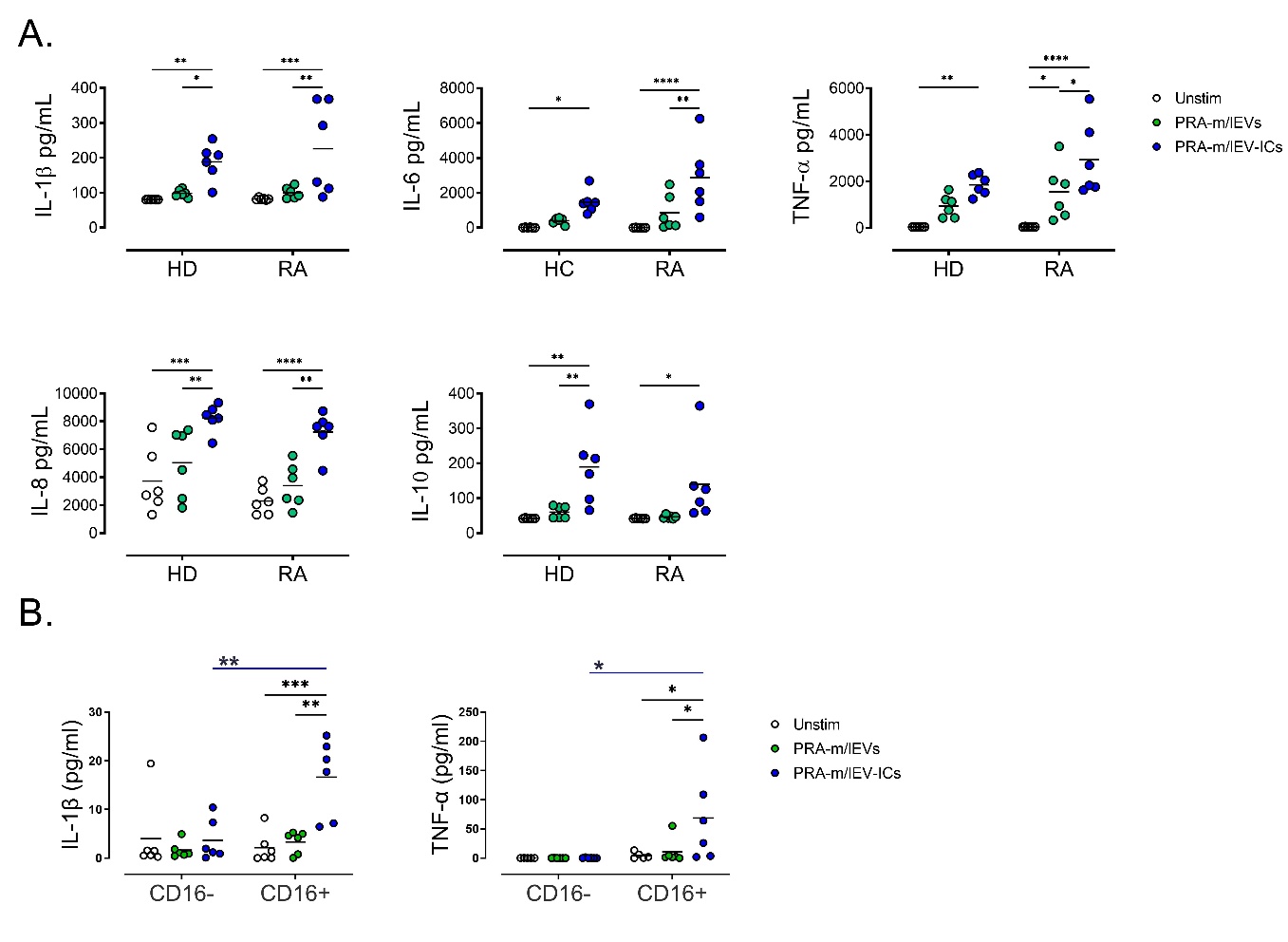


**Figure S6.** **Effect of m/lEVs on cytokine production by macrophages and subsets of monocytes. A.** Levels of IL-1β, IL-6, IL-8, IL-10, and TNF-α in the supernatants of monocyte-derived macrophages (MDM) from HD and patients with RA exposed or not to PRA-m/lEVs and PRA-m/lEV-ICs for 6 h. **B.** Levels of IL-1β and TNF-α in the supernatants of CD16^−^ and CD16^+^ monocytes cultured for 24 h in absence or presence of PRA-m/lEVs and PRA-m/lEV-ICs. Two-way ANOVA test with Šidák post-test. * p<0.05, ** p<0.01, *** p<0.001, **** p<0.0001.
